# TRPC channels regulate Ca^2+^-signaling and short-term plasticity of fast glutamatergic synapses

**DOI:** 10.1371/journal.pbio.3000445

**Published:** 2019-09-19

**Authors:** Yvonne Schwarz, Katharina Oleinikov, Barbara Schindeldecker, Amanda Wyatt, Petra Weißgerber, Veit Flockerzi, Ulrich Boehm, Marc Freichel, Dieter Bruns

**Affiliations:** 1 Institute for Physiology, Saarland University, CIPMM, Homburg/Saar, Germany; 2 Experimental and Clinical Pharmacology and Toxicology, PZMS, Saarland University, Homburg/Saar, Germany; 3 Institute of Pharmacology, University of Heidelberg, Heidelberg, Germany; Yale University, UNITED STATES

## Abstract

Transient receptor potential (TRP) proteins form Ca^2+^-permeable, nonselective cation channels, but their role in neuronal Ca^2+^ homeostasis is elusive. In the present paper, we show that TRPC channels potently regulate synaptic plasticity by changing the presynaptic Ca^2+^-homeostasis of hippocampal neurons. Specifically, loss of TRPC1/C4/C5 channels decreases basal-evoked secretion, reduces the pool size of readily releasable vesicles, and accelerates synaptic depression during high-frequency stimulation (HFS). In contrast, primary TRPC5 channel-expressing neurons, identified by a novel TRPC5–τ-green fluorescent protein (τGFP) knockin mouse line, show strong short-term enhancement (STE) of synaptic signaling during HFS, indicating a key role of TRPC5 in short-term plasticity. Lentiviral expression of either TRPC1 or TRPC5 turns classic synaptic depression of wild-type neurons into STE, demonstrating that TRPCs are instrumental in regulating synaptic plasticity. Presynaptic Ca^2+^ imaging shows that TRPC activity strongly boosts synaptic Ca^2+^ dynamics, showing that TRPC channels provide an additional presynaptic Ca^2+^ entry pathway, which efficiently regulates synaptic strength and plasticity.

## Abstract 2

Transient receptor potential (TRP) proteins can form non-selective cation channels, but their role in synaptic transmission is poorly understood. This study shows that calcium-permeable TRPC channels provide an additional calcium entry pathway at presynaptic sites and are efficient regulators of synaptic strength and plasticity.

## Introduction

Short-term presynaptic plasticity is a widespread and important means of synaptic regulation, but the underlying mechanisms are not fully understood [1,2]. Calcium plays an important role in many use-dependent forms of plasticity, and STE of synaptic signaling can be changed by calcium channel facilitation, saturation of endogenous calcium buffers, or other means downstream of the voltage-gated calcium channel (VGCC)-mediated Ca^2+^ entry [2]. The canonical transient receptor potential channel (TRPC) family comprises 7 members (TRPC1 to TRPC7) that are able to form Ca^2+^-permeable nonselective cation channels [3]. TRPC channels can be activated in response to stimulation of phospholipase C-coupled receptors [4], subsequent to store depletion [5–7], or directly by intracellular Ca^2+^ [8–10]. They have been implicated in diverse neuronal functions, such as excitability [11–13], excitotoxicity [14], neurogenesis [15], and neurite outgrowth [16–18]. Based on phylogenetic analyses and sequence similarities, the TRPC subfamily can be divided into 3 subgroups: TRPC1/TRPC4/TRPC5, TRPC3/TRPC6/TRPC7, and TRPC2 [15,19]. Group-specific interactions between TRPC1, TRPC4, and TRPC5, but not with members of the TRPC3/TRPC6/TRPC7 subgroup, were observed in heterologous expression systems [20]. Furthermore, *Trpc1*, *Trpc4*, and *Trpc5* genes were found to be co-expressed in subregions of the hippocampus, as documented by immunohistochemistry and in situ hybridization [21–25]. TRPC4 and TRPC5 are strongly potentiated [9,26] or even directly activated by elevations of intracellular calcium concentration ([Ca]i) [8]. The latter study demonstrated that Ca^2+^ entry through other channels, including VGCCs, directly enhanced TRPC5 activity. In contrast, TRPC1 channels do not respond to intracellular Ca^2+^ elevations but are inserted into the plasma membrane in response to local Ca^2+^ entry through Orai1 channels [27].

Although TRPC1, TRPC4, and TRPC5 have been implicated in the generation of Ca^2+^ signals in different secretory cells [16,18,21,25,28,29], their potential contribution to Ca^2+^-dependent transmitter release and neuronal communication has remained largely controversial [13,30–36]. In a recent study, we showed that genetic loss of TRPC1, TRPC4, and TRPC5 channels (TRPC triple knockout [tko]) impairs working memory and flexible relearning, most likely by reducing synaptic transmission of hippocampal neurons [37]. Yet, the underlying molecular mechanisms of how these TRPC channels are able to regulate synaptic signaling have remained unclear.

Here, we delineate so far unprecedented TRPC actions in the regulation of synaptic vesicle release. Using electrophysiological recordings of hippocampal neurons from TRPC-deficient mice and from a novel Cre knock-in mouse line, which genetically labels primary TRPC5-expressing neurons, along with presynaptic Ca^2+^ measurements and lentiviral TRPC expression experiments, we provide first evidence that TRPC5 channel activity efficiently regulates presynaptic Ca^2+^-dynamics and controls synaptic vesicle recruitment. Collectively, our data show that TRPC5 channels play a pivotal role in the regulation of short-term synaptic plasticity in neurons.

## Results

### Loss of TRPC1/C4/C5 reduces the replenishment rate and the readily releasable pool size of vesicles

**Fig 1:**
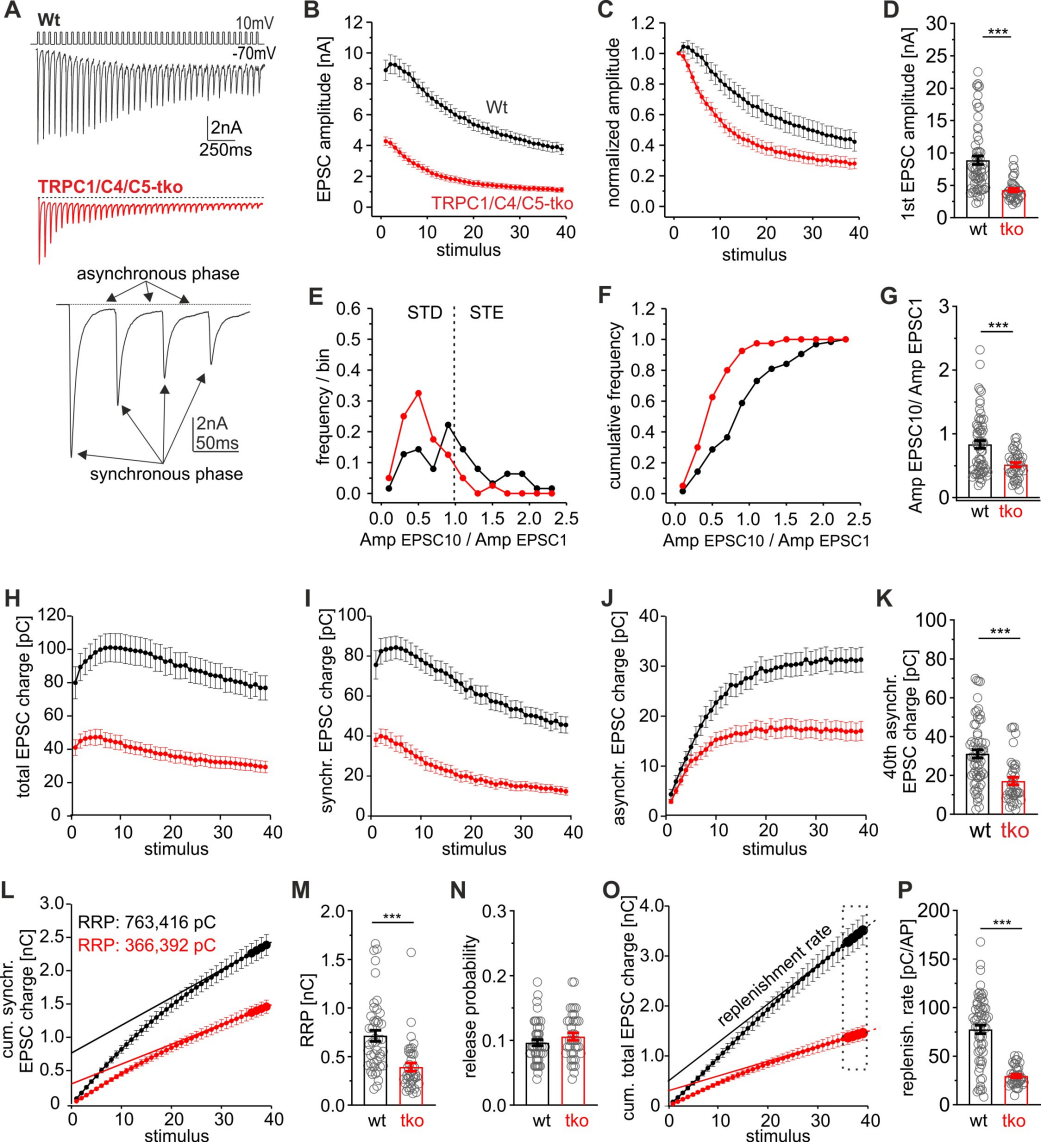
TRPC channels modulate the vesicle replenishment rate and the RRP size. (A) Exemplary EPSCs triggered by HFS (20 Hz, 40 AP/2 s) of autaptic wt and TRPC1/C4/C5 tko neurons (inset magnified view on the first 4 EPSCs of the train response. Arrows depict the synchronous and asynchronous phase of secretion). (B and C) TRPC deficiency reduces the EPSC amplitudes and accelerates the time course of synaptic depression (In panel C, data were normalized to the first EPSC amplitude). (D) The first EPSC amplitude is significantly reduced in tko cells (wt, *n* = 63; tko, *n* = 39 cells; *p* = 1.9 × 10^−9^). (E and F) The Amp_EPSC10_/Amp_EPSC1_ ratios of wt neurons range from STD to STE. Loss of TRPCs shifts the Amp_EPSC10_/Amp_EPSC1_ ratio towards STD. (G) The Amp_EPSC10_/Amp_EPSC1_ ratio is reduced in tko cells (*p* = 0.00067). (H, I, and J) The total synchronous and asynchronous charges are smaller in tko cells (red) when compared with controls (black). (K) Quantification of the 40th asynchronous charge (*p* = 0.0000056). (L) Mean cumulative synchronous release components during the 20-Hz train. Continuous line, linear regression of the last 5 data points back-extrapolated to stimulus = 0 to estimate the initial RRP size. Note that the RRP analysis was confined to wt neurons that reached a steady-state response in the late phase of stimulus train (45 out of 63 neurons). (M) The RRP size is significantly decreased in tko cells (wt, *n* = 45; tko, *n* = 39; *p* = 0.0000013). N. The release probability remained unchanged between groups (*p* = 0.2). (O) Cumulative total charge during 20-Hz train stimulation. The slope of the linear regression from the last 4 stimulation points (continuous line) rendered an estimate of the replenishment rate in panel P. (P) The replenishment rate is significantly reduced in tko neurons (*p* = 1.6 × 10^−10^). **p < 0.01; ***p < 0.001; statistical significance was assessed by Mann-Whitney rank sum test. Underlying data can be found in S1 Data. Amp, amplitude; AP, action potential; EPSC, evoked postsynaptic current; HFS, high-frequency stimulation; RRP, readily releasable pool; STD, short-term depression; STE, short-term enhancement; tko, triple knockout; TRPC, transient receptor potential canonical; wt, wild type.

To elucidate the mechanisms how genetic loss of TRPC1/C4/C5-channels affects the action potential (AP)-evoked postsynaptic current (EPSC), we used high-frequency train stimulations (20 Hz for 2 s) in autaptic hippocampal wild-type (wt) and TRPC1/C4/C5 tko neurons. Synaptic responses were analyzed with respect to the readily releasable pool (RRP) size, release probability (Pr), paired-pulse ratio (PPR), replenishment rate, and synaptic depression. Loss of TRPC channels significantly reduced the amplitude of the first EPSC during the train (Fig 1A, 1B and 1D) and clearly accelerated the time course of synaptic depression when compared with controls (Fig 1C). Although wt cells showed a clear heterogeneity in synaptic signaling ranging from short-term enhancement (STE, Amp_EPSC10_/Amp_EPSC1_ ratio >1) to short-term depression (STD; Amp_EPSC10_/Amp_EPSC1_ ratio <1) during HFS, tko neurons predominantly displayed STD (Fig 1E–1G). Furthermore, the synchronous and asynchronous phase of the total synaptic charge transfer were similarly diminished (Fig 1H–1K), indicating that factors upstream of the RRP are affected by genetic loss of the TRPCs. To determine the RRP size, the data plot of the cumulative synchronous EPSC charge was approximated with a linear regression fitting the last 5 stimuli [38] (Fig 1L). Back-extrapolating the linear component of the steady-state phase renders an estimate of the initial RRP size (Fig 1L). Indeed, loss of TRPC channels strongly reduced the RRP size (Fig 1M) but left the Pr (i.e., first EPSC charge divided by the RRP charge) unchanged (Fig 1N). To determine the replenishment rate of vesicles during pool depletion, the slope of the plot of the cumulative total charge was approximated by linear regression fitting over the last 5 stimuli (Fig 1O). The replenishment rate was strongly reduced in tko neurons compared with controls (Fig 1P). Taken together, these results suggest that TRPC channels influence vesicle recruitment and thereby regulate the RRP size and the time course of synaptic depression during HFS.

To study the subcellular distribution of TRPC channels, we co-immunolabeled neuronal cultures with antibodies directed against TRPC5 and the presynaptic marker protein bassoon. Confocal imaging revealed clear TRPC5 staining in somatic regions and axonal branches (S1 Fig, upper panels) as well as in varicosity-like thickenings of fine axonal branches, where it colocalized with bassoon (S1 Fig, lower panels). The specificity of the TRPC labeling was verified in cultures of TRPC1/C4/C5-tko neurons, which did not exhibit a discernable TRPC5 immunosignal (S1B Fig). TRPC5 was detected in about half of the presynaptic terminals (positive for bassoon, S1E Fig), indicating a heterogeneous TRPC5 expression in hippocampal neurons. In any case, these results are in line with our functional analyses showing that TRPC activity alters synaptic efficacy.

### Primary TRPC5-expressing neurons show strong STE of synaptic signaling

To identify primary TRPC5-expressing neurons by genetic labeling, we generated a new TRPC5-Cre knockin (KI) mouse strain (TRPC5-internal ribosomal entry site cre recombinase [IC], S1A and S1B Fig). Here, the *trpc5* gene is followed by an internal ribosome entry site (IRES) and by a Cre recombinase cDNA. When crossbred with ROSA26-floxed-stop-τGFP (eR26-τGFP) reporter mice [39], the Cre recombinase excises a floxed termination sequence 5ʹ to the τGFP transgene within the *ROSA26* locus resulting in constitutive reporter expression in TRPC5-expressing cells.

**Fig 2:**
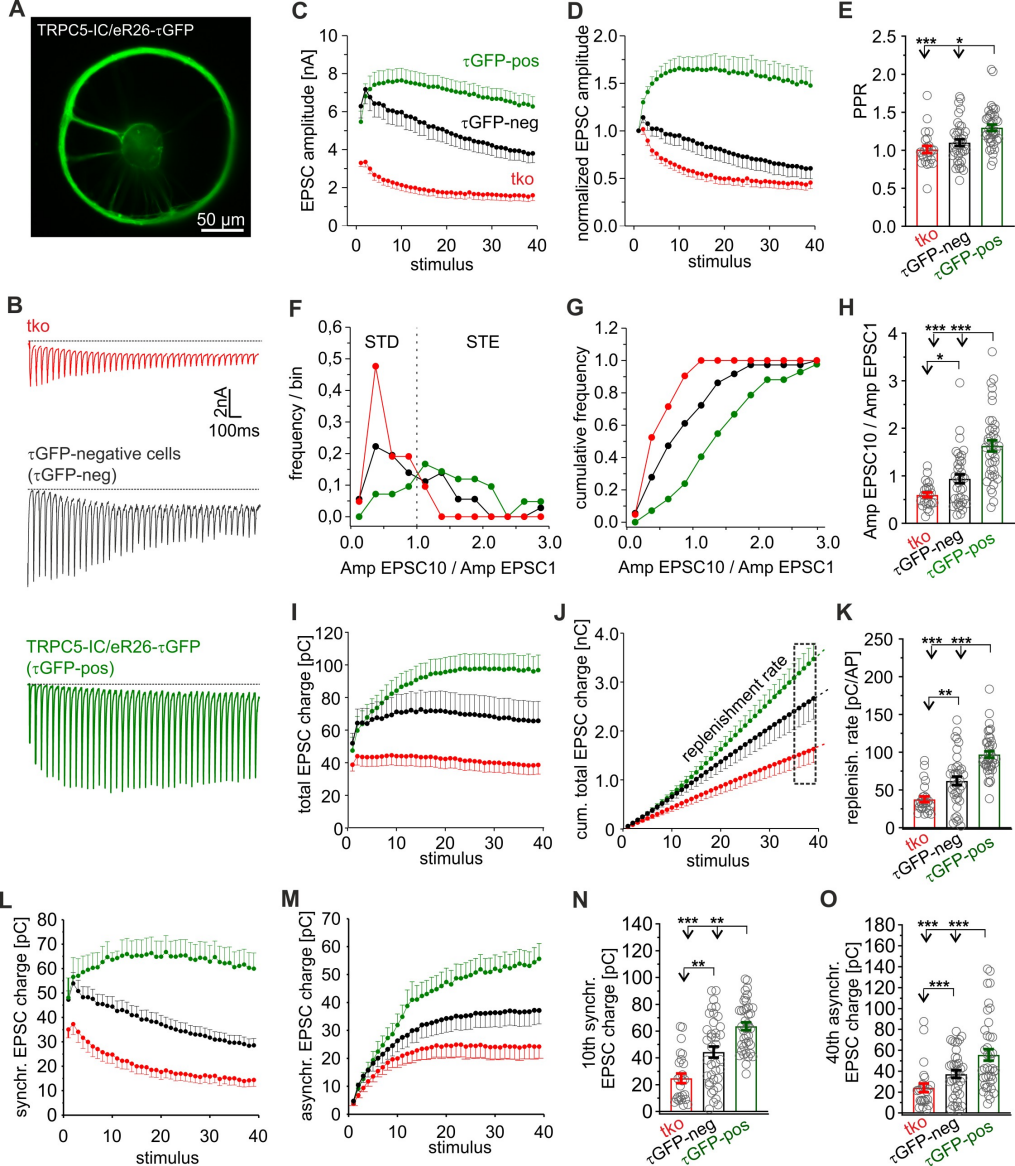
Endogenous TRPC5 channels promote STE of synaptic signaling. (A) Exemplary epifluorescence image of an autaptic TRPC5-IC/eR26-τGFP neuron. (B) Sample recordings from tko, τGFP-negative and τGFP-positive neurons upon 20-Hz HFS. (C–E) τGFP-positive cells show a strong STE of synaptic signaling and an increased PPR (panel E, EPSC2/EPSC1) when compared with tko or τGFP-negative neurons (τGFP-positive versus tko *p* = 0.00005; τGFP-positive versus τGFP-negative *p* = 0.003). (F and G) Frequency and cumulative frequency distribution of the Amp_EPSC10_/Amp_EPSC1_ ratios for tko (red), τGFP-negative (black), and τGFP-positive neurons (green). (H) The Amp_EPSC10_/Amp_EPSC1_ ratio is higher in τGFP-negative cells than in tko neurons and even further increased in τGFP-positive cells (τGFP positive versus tko *p* = 7.892264 × 10^−9^; τGFP positive versus τGFP negative *p* = 0.0009; τGFP negative and tko *p* = 0.02). (I) Time course of total charge during HFS. (J and K) The replenishment rate determined from the cumulative charge plot shown in panel J gradually increases between tko, τGFP-negative, and τGFP-positive neurons (τGFP positive versus tko *p* = 1.5 × 10^−10^; τGFP positive versus τGFP-negative *p* = 0.0001; τGFP negative and tko *p* = 0.002). (L and M) Time courses of synchronous and asynchronous charge transfer during the stimulus train. (N and O) The 10th EPSC charge and the 40th asynchronous charge are significantly increased in τGFP-negative and -positive neurons (10th charge: τGFP positive versus tko *p* = 1.7 × 10^−8^; τGFP positive versus τGFP negative *p* = 0.003; τGFP negative and tko *p* = 0.002; 40th charge: τGFP positive versus tko *p* = 0.00004; τGFP poisitive versus τGFP negative *p* = 0.004; τGFP negative and tko *p* = 0.018). Data were collected from tko (*n* = 25), τGFP-negative (*n* = 38), and τGFP-positive (*n* = 40) neurons. *p < 0.05, **p < 0.01, ***p < 0.001, one-way ANOVA on ranks followed by Dunn’s post hoc test; significance was also tested by Mann-Whitney rank sum test for τGFP-negative cells versus tko. Underlying data can be found in S1 Data. Amp, amplitude; AP, action potential; EPSC, evoked postsynaptic current; eR26, ROSA26-floxed-stop; HFS, high-frequency stimulation; IC, internal ribosomal entry site cre recombinase; PPR, paired-pulse ratio; STD, short-term depression; STE, short-term enhancement; tko, triple knockout; TRPC, transient receptor potential canonical; τGFP, τ-green fluorescent protein.

About 8% of the hippocampal neurons prepared from TRPC5-IC/eR26-τGFP mice were found to be τGFP-positive (7.7% ± 1.5% of 831 total cells, 2 preparations) and showed a clear immunosignal for TRPC5 (S2C and S2D Fig). Yet, among neurons, which were negative for τGFP, we also observed TRPC5-expressing cells (38.5% ± 3.58% of the τGFP-negative neurons; S2D Fig), most likely because the coding sequence 3ʹ of the IRES sequence is known to be expressed at significantly lower levels than the coding sequence 5ʹ of the IRES [40]. A similar frequency of TRPC5 positive cells was found with immunolabeling cultures of wt neurons (52% ± 3%, 195 out of 375 cells, 3 preparations), showing that IRES insertion does not affect the expression of the upstream protein (S2E and S2F Fig). Thus, hippocampal neurons heterogeneously express TRPC5 and τGFP labeling (in cultures of KI mutant mice) enables the identification of neurons that reliably express TRPC5. We comparatively analyzed synaptic signaling from τGFP-positive and τGFP-negative neurons with that of tko neurons. Indeed, τGFP-positive (i.e., TRPC5-expressing) neurons showed strong STE of their synaptic response during HFS and an increased PPR (Fig 2A–2E). In contrast, tko neurons started off with lower first EPSC amplitude and showed classical STD during HFS. The Amp_EPSC10_/Amp_EPSC1_ ratio is altered for the entire population of τGFP-positive neurons (Fig 2F and 2G), reaching on average a nearly 3-fold higher ratio than in tko neurons (τGFP-positive neurons: 1.63 ± 0.11; tko neurons 0.6 ± 0.05, *p* < 0.001, one-way ANOVA on ranks, Fig 2H). Furthermore, the synchronous and asynchronous charge component of the evoked response and the replenishment rate recorded in τGFP-positive cells were strongly enhanced when compared with synaptic responses of tko neurons (Fig 2I–2O). τGFP-negative neurons, instead, displayed an intermediate plasticity phenotype with respect to Amp_EPSC10_/Amp_EPSC1_ ratio (0.94 ± 0.09), total synaptic charge transfer, synchronous charge, and asynchronous charge as well as PPR and replenishment rate (Fig 2). These results agree with the observation that about 39% of the τGFP-negative neurons express TRPC5 (S2 Fig). Overall, the systematic changes in STP between τGFP-positive and -negative neurons as well as tko neurons correlate well with the differential expression of TRPC5 in these groups, indicating that endogenous TRPC5 activity is a key factor in regulating synaptic plasticity.

### TRPC activity promotes presynaptic short-term plasticity in hippocampal neurons

**Fig 3:**
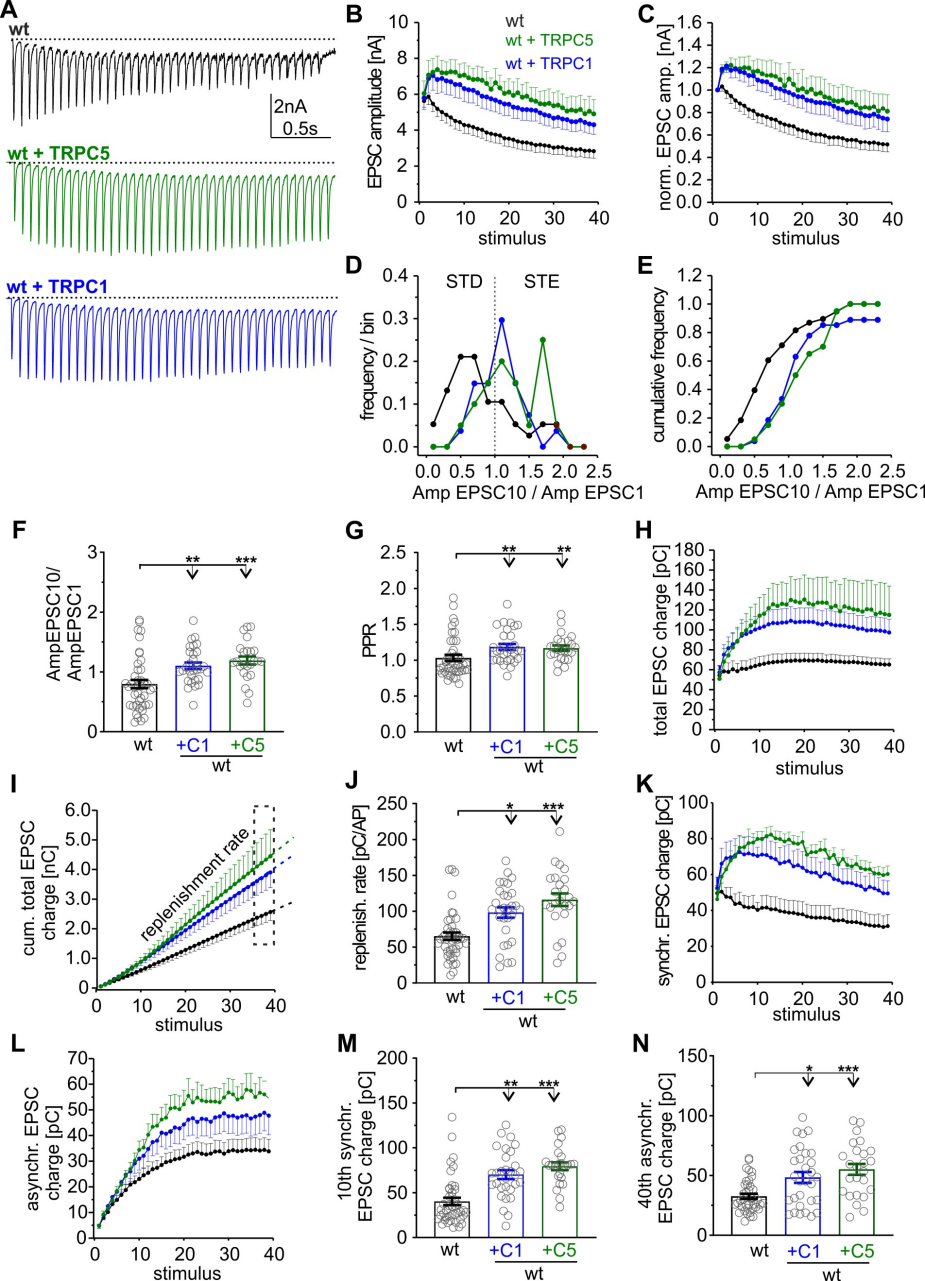
Expression of either TRPC1 or TRPC5 in wt neurons turns synaptic depression into STE. (A) Sample recordings of EPSCs triggered by HFS (20 Hz, 40 AP/2 s) of autaptic wt neurons and those expressing TRPC5 or TRPC1. (B) Activity-dependent increase in the EPSC amplitudes in wt cells expressing C1 or C5. (C) TRPC expression causes STE of synaptic signaling (data were normalized to the initial peak EPSC amplitude). (D and E) Expression of TRPC1 or TRPC5 shifts the frequency distribution of the Amp_EPSC10_/Amp_EPSC1_ ratios to higher values. (F) Mean Amp_EPSC10_/Amp_EPSC1_ ratio for the indicated groups (+C1, *p* = 0.002; +C5, *p* = 0.0001; versus wt). (G) Mean paired pulse ratio (+C1, *p* = 0.0036; +C5, *p* = 0.00585; versus wt). (H) Time course of total charge transfer. (I) Mean cumulative total charge transfer during HFS. The replenishment rate was determined from the slope of the cumulative plot (last 4 data points). (J) The replenishment rate is significantly enhanced in cells expressing either C1 or C5 (+C1, *p* = 0.003; +C5, *p* = 0.00001; versus wt). (K and L) Time courses of synchronous (K) and asynchronous charge transfer (L) during the stimulus train. (M and N) The 10th EPSC charge (M) and the 40th asynchronous charge (N) are significantly increased with TRPC expression (10th charge: +C1, *p* = 0.00001; +C5, *p* = 0.0000001; versus wt; 40th asynchronous: +C1, *p* = 0.02; +C5, *p* = 0.0005; versus wt). Data were collected from the following number of cells: wt, *n* = 43; wt + TRPC1, *n* = 30; wt + TRPC5, *n* = 24; *p < 0.05; **p < 0.01; ***p < 0.001; one-way ANOVA on ranks followed by Dunn’s post hoc test. Underlying data can be found in S1 Data. Amp, amplitude; AP, action potential; EPSC, evoked postsynaptic current; HFS, high-frequency stimulation; PPR, paired-pulse ratio; STD, short-term depression; STE, short-term enhancement; TRPC, transient receptor potential canonical; wt, wild type.

To further study the impact of TRPC activity on synaptic signaling, we expressed TRPC1 or TRPC5 in wt neurons using lentiviral transduction. Wt neurons expressing either TRPC1 or TRPC5 developed a robust STE of the synaptic response during the stimulation train (Fig 3A–3C). TRPC channel expression shifts the frequency distribution for Amp_EPSC10_/Amp_EPSC1_ ratios to higher values, confirming general changes in STP (Fig 3D–3F). Starting from an unchanged first AP-evoked response (wt: 5.61 ± 0.56 nA; wt + C1: 5.8 ± 0.65 nA; wt + C5: 6.04 ± 0.69 nA; Fig 3B), TRPC expression significantly increased the PPR and elevated the synchronous as well as asynchronous charge transfer when compared with controls (Fig 3G–3N). Overall, a 1.5- to 2-fold higher total synaptic charge transfer (wt: 65.2 ± 5.8 pC; wt + C1: 98.27 ± 7.20 pC; wt + C5: 116.01 ± 8.79 pC) and a corresponding increase in the vesicle supply rate was observed (Fig 3H–3J). Importantly, the synaptic plasticity phenotype observed with lentiviral TRPC5 expression largely mimics that of the τGFP-positive neurons (compare with Fig 2), demonstrating that TRPC5 channels are instrumental in controlling synaptic plasticity.

The short-term plasticity (STP) phenotype of TRPC-expressing cells often hindered the RRP determination because a steady-state response in the late phase of stimulus train was not reached [41]. Therefore, we made use of hypertonic sucrose stimulation to provide an estimate of the RRP size and the Pr with single AP stimulation [42]. Consistent with our results obtained with HFS, loss of TRPC1/C4/C5 reduced the AP-evoked response and the RRP size but left the Pr unchanged (S3 Fig). Furthermore, neither the expression of TRPC5 nor of TRPC1 in wt neurons significantly altered the RRP and the Pr (S3C Fig). Thus, TRPC activity does not affect the basal release probability but rather enhances the vesicle recruitment in an activity-dependent manner during HFS.

Given that TRPC channels have been implicated in growth cone guidance and morphology [16,17], one might speculate that TRPC-mediated changes of synaptic signaling may at least in part be due to alterations in the number of synapses. To pursue this issue, we immunolabeled autaptic wt, tko, and wt neurons expressing either TRPC variant with the presynaptic marker protein bassoon (S4 Fig). Yet, synaptogenesis was neither affected by loss of TRPC channels nor by their lentiviral expression. To study whether TRPC channels interfere with the overall synaptic structure, cultured hippocampal neurons were co-immunolabeled for the active zone protein bassoon and the postsynaptic density protein PSD-95. Because bassoon and PSD-95 reside on either side of the synaptic cleft, we determined the degree of juxtaposition (or colocalization) of both signals up to a distance of 770 nm from the bassoon signal (S4C Fig). Quantitation of the percentage of “bassoon area” covered by PSD-95 signal revealed that neither the absence of TRPC1/4/5 nor the expression of TRPC1 or C5 in wt neurons altered the overall colocalization of bassoon and PSD95 puncta (S4D and S4E Fig). Taken together, these results render the possibility unlikely that changes in synapse number or organization contribute to the observed TRPC-mediated alterations in synaptic signaling.

### TRPC channel activity promotes efficient recovery from synaptic depression

To further explore the role of synaptic TRPC channels in vesicle replenishment, we analyzed the recovery kinetics of phasic release from depression after HFS (S5 Fig). The degree of recovery was determined by dividing the EPSC amplitude of the test pulse (given at various time intervals after HFS) by that of the first response during the stimulus train (20 Hz; S5A and S5B Fig). A single exponential function was used to approximate the time course of recovery (see S5 Fig legend for details). The results show that expression of TRPC1 or TRPC5 in wt neurons diminished synaptic depression during the train (S5C Fig), accelerated the recovery kinetics (S5D Fig), and strongly augmented the EPSC amplitude when compared with controls (S5E Fig). In contrast, loss of TRPC activity (tko neurons) slowed down the recovery kinetics from synaptic depression when compared with controls (S5F–S5H Fig). Collectively, these results indicate that activation of synaptic TRPC channels leads to more efficient and faster mobilization of vesicles from the reserve pool.

### Buffering presynaptic [Ca]i abolishes TRPC-dependent STE

**Fig 4:**
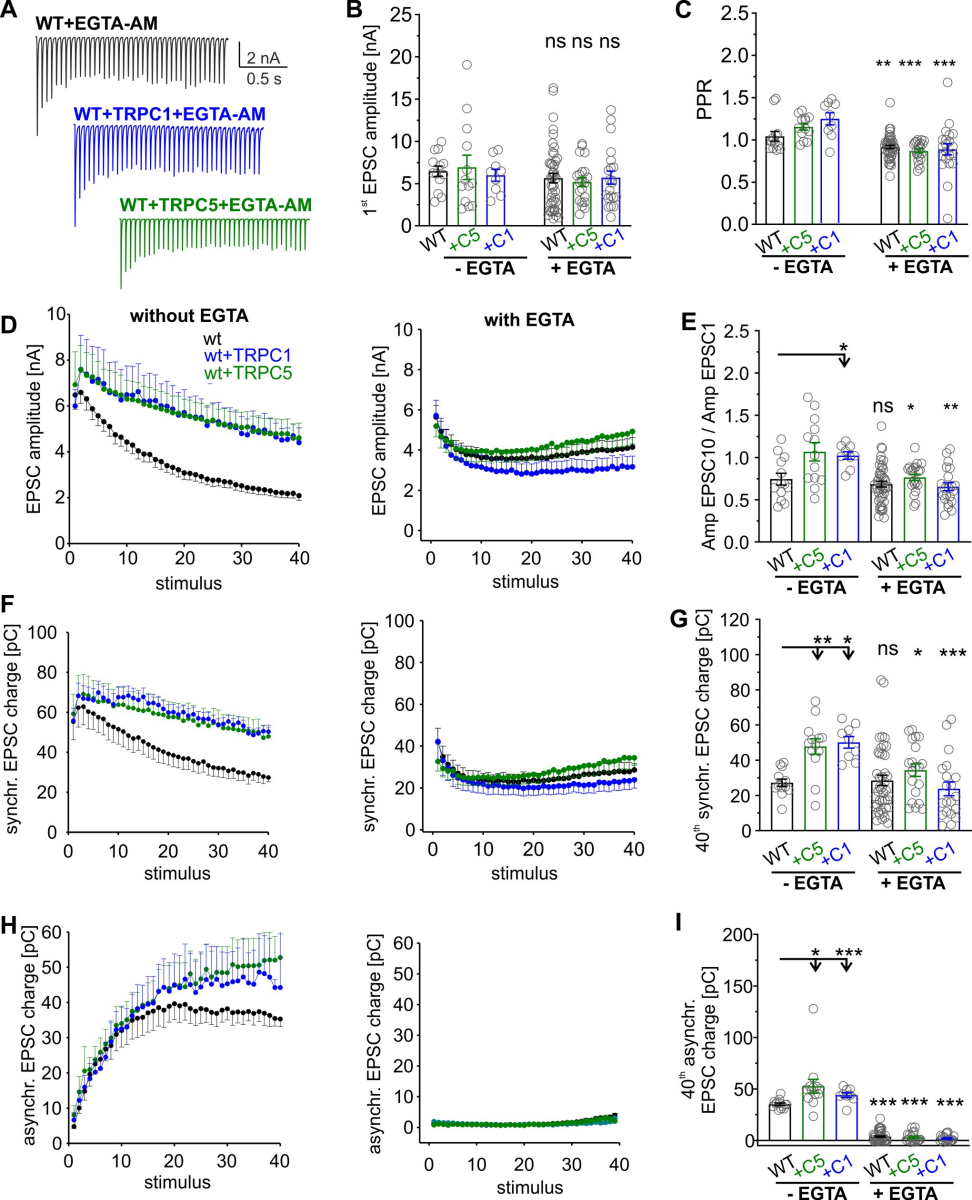
Buffering presynaptic [Ca]i abolishes TRPC-dependent short-term facilitation. (A) Representative EPSC traces for wt neurons and those expressing TRPC1 or TRPC5 after EGTA-AM treatment. EGTA abolishes asynchronous secretion causing a phase of steady-state synchronous secretion and prevents the TRPC-mediated STE of synaptic signaling. (B). Neither TRPC expression (C1 or C5) nor pretreatment with EGTA affects the first EPSC amplitude of the train response (between groups without EGTA: *p* = 0.7; in-group with versus without EGTA: wt, *p* = 0.48; +C1, *p* = 0.49; +C5, *p* = 0.67). (C) EGTA significantly reduces the PPR for all groups (in-group with versus without EGTA: wt, *p* = 0.03; +C1, *p* = 0.001; +C5, *p* = 2 × 10^−6^). (D, F, H) Activity-dependent changes of EPSC amplitude (D), synchronous release (F), and asynchronous release (H) for wt neurons and those expressing TRPC1 or TRPC5 without (left panels) and with EGTA treatment (right panels). (E and G) The TRPC-mediated increase in the Amp_EPSC10_/Amp_EPSC1_ ratio (E) and the 40^th^ synchronous EPSC charge (panel G, last pulse of the train shown in panel F) are prevented with EGTA treatment (EPSC10/EPSC1 [between groups without EGTA]: +C1, *p* = 0.07; +C5, *p* = 0.05, versus wt; in-group with versus without EGTA: wt, *p* = 0.5; +C1, *p* = 0.00004; +C5, *p* = 0.028; 40th synchronous [between groups without EGTA]: +C5, *p* = 0.002, +C1, *p* = 0.0014 versus wt; in-group with versus without EGTA: wt, *p* = 0.36; +C1, *p* = 0.0004; +C5, *p* = 5.8 × 10^−9^). (H and I) EGTA treatment diminishes asynchronous secretion in all groups, indicating effective buffering of presynaptic [Ca]i (+C5, *p* = 0.0008, +C1, *p* = 0.05; in-group with versus without EGTA: wt, *p* = 1.4 × 10^−12^; +C1, *p* = 0.0000003; +C5, *p* = 5.8 × 10^−9^). Data was collected from EGTA-treated wt (*n* = 45), wt + TRPC1 (*n* = 21), and wt + TRPC5 (*n* = 21) cells and nontreated wt (*n* = 13), wt + TRPC1 (*n* = 9), and wt + TRPC5 (*n* = 13) cells. *p < 0.05, one-way ANOVA on ranks followed by Dunn’s post hoc test for groups without EGTA; **p < 0.01, ***p < 0.001, Mann-Whitney U rank sum test for the in-group comparison with and without EGTA treatment. Underlying data can be found in S1 Data. EGTA-AM, ethylene glycol-bis(β-aminoethyl ether)-N,N,N′,N′-tetraacetic acid acetoxymethyl ester; EPSC, evoked postsynaptic current; ns, not significant; PPR, paired-pulse ratio; STE, short-term enhancement; TRPC, transient receptor potential canonical; wt, wild type.

Using photolytic uncaging experiments, we have previously shown that TRPC5 channels can directly be activated by an intracellular Ca^2+^ increase at the millisecond time scale [8]. Thus, it is possible that rapid activation of TRPC-mediated Ca^2+^ permeabilities further elevate the presynaptic Ca^2+^ rise during the stimulus train. To minimize the accumulation of intraterminal calcium during HFS, neuronal cultures were preincubated with the membrane-permeable calcium chelator ethylene glycol-bis(β-aminoethyl ether)-N,N,N′,N′-tetraacetic acid acetoxymethyl ester (EGTA-AM; 300 μM for 5 min; Fig 4). Owing to its slow kinetics and high affinity, EGTA buffers global Ca^2+^ without affecting phasic transmitter release [1]. EGTA did not change the first EPSC amplitude but significantly reduced the PPR of responses for wt neurons and those expressing additional TRPC1 or TRPC5 (Fig 4A, 4B, and 4C). In the same line, EGTA nearly abolished the delayed asynchronous secretion (Fig 4H and 4I) and caused steady-state phasic release (Fig 4D and 4F right panels; see also [38]), indicating efficient buffering of residual [Ca]i in our experiments. Importantly, wt neurons expressing either TRPC variant failed to promote any STE of synaptic signaling in the presence of EGTA (Fig 4D–4G). Both the Amp_EPSC10_/Amp_EPSC1_ ratio (Fig 4E) and the synchronous charge (Fig 4G) were significantly reduced when compared with the nontreated controls. These results indicate that enhanced buffering of global [Ca]i by EGTA abolishes the TRPC-mediated STE of synaptic signaling. Collectively, our results support a model wherein TRPC channels are rapidly activated by elevated levels of bulk [Ca]i (in response to Ca^2+^ entry through presynaptic voltage-gated Ca^2+^ channels) and in turn may amplify and prolong the presynaptic Ca^2+^ rise during HFS, thereby increasing the Ca^2+^ dependent rates of vesicle replenishment [43–45].

### Homomeric TRPC5 channels promote STE of synaptic signaling

**Fig 5:**
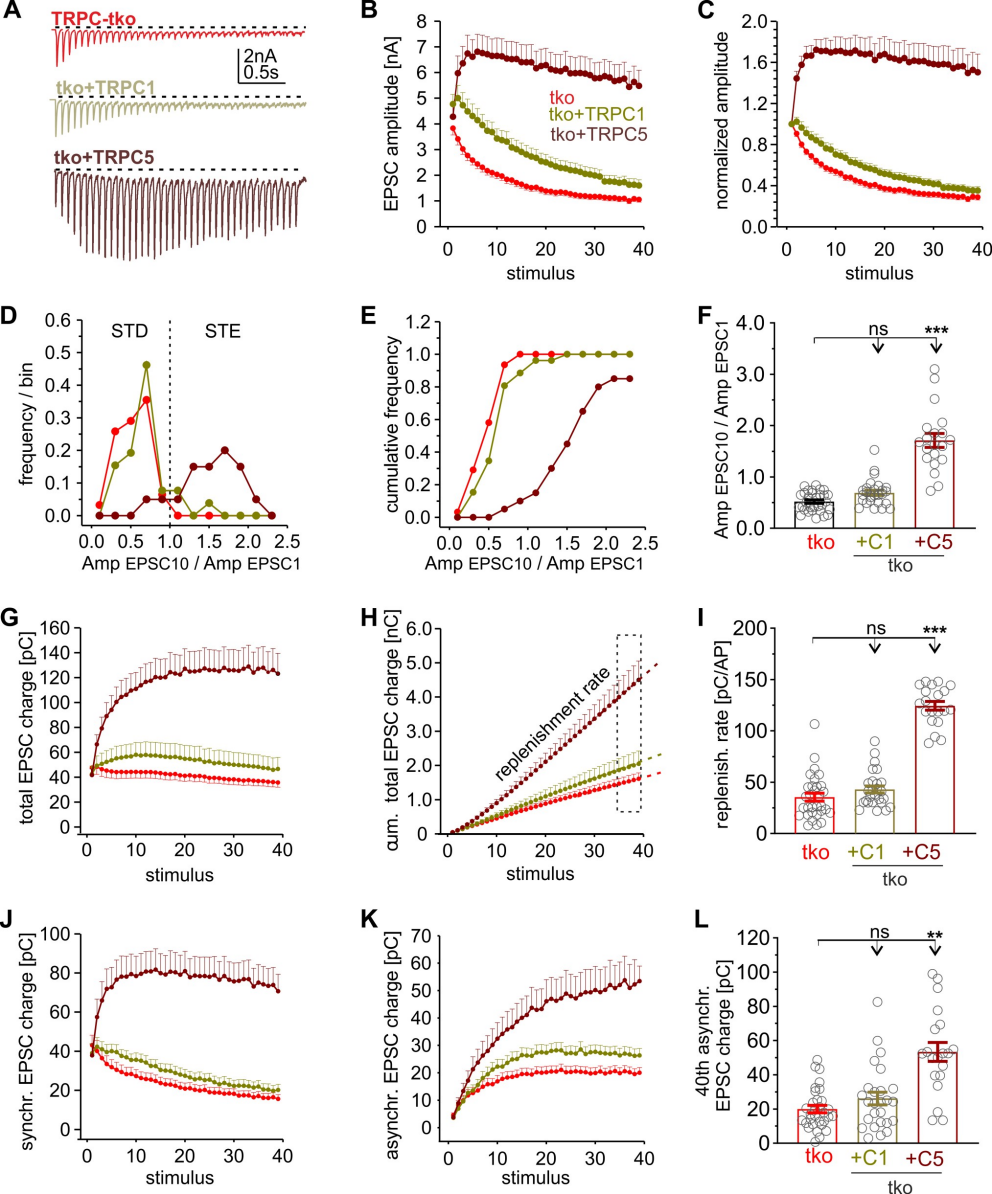
Homomeric TRPC5 channels promote strong STE of synaptic signaling in tko neurons. (A) Sample recordings of EPSCs triggered by HFS (20 Hz, 40 AP/2 s) of tko neurons and those expressing C1 or C5. (B) Starting from similar initial EPSC amplitudes, only TRPC5 expression causes STE of the synaptic response. (C) EPSC amplitude changes during HFS normalized to the amplitude of the first response. (D–F) The Amp_EPSC10_/Amp_EPSC1_ ratio of TRPC5-expressing tko neurons is shifted to the STE range and nearly 3-fold higher compared with tko and tko + C1 responses (panel F, +C1, *p* = 0.14; +C5, *p* = 1.9 × 10^−10^, versus tko). (G–I) TRPC5, but not TRPC1, expression elevates the total synaptic charge transfer and increases the replenishment rate (+C1 *p* = 0.1; +C5, *p* = 2 × 10^−10^, versus tko). (J and K) Time courses of synchronous (J) and asynchronous charge transfer (K) during the stimulus train. (L) The 40th asynchronous charge is significantly increased with TRPC5 expression (+C1, *p* = 0.98; +C5, *p* = 0.000005; versus tko). Data were collected from the following number of neurons: tko, *n* = 31; tko + TRPC1, *n* = 26; tko + TRPC5, *n* = 20; **p < 0.01, ***p < 0.001; one-way ANOVA on ranks followed by Dunn’s post hoc test. Underlying data can be found in S1 Data. Amp, amplitude; AP, action potential; EPSC, evoked postsynaptic current; HFS, high-frequency stimulation; ns, not significant; STD, short-term depression; STE, short-term enhancement; tko, triple knockout; TRPC, transient receptor potential canonical.

TRPC4 and TRPC5 are strongly potentiated by elevation of [Ca]i [9] and can functionally couple to the Ca^2+^ entry through voltage-gated Ca^2+^ channels [8,26]. In contrast, no such regulation has been reported for TRPC1 [27], appearing contradictory to our finding that TRPC1 caused strong STE during repetitive stimulation. To address this issue, we compared the functional consequences of TRPC1 and TRPC5 expression in tko neurons (Fig 5). Expression of TRPC5 caused a strong STE of synaptic signaling (Fig 5A, 5B and 5C) and reproduced the phenotype seen with τGFP-positive wt neurons (Fig 2). TRPC5 expression significantly increased the PPR (tko: 0.9 ± 0.03, tko + C5: 1.44 ± 0.07; *p* < 0.001, one-way ANOVA on ranks). It shifted the distribution of Amp_EPSC10_/Amp_EPSC1_ ratios (mean ± SEM: 1.71 ± 0.14) toward the STE range (Fig 5D, 5E and 5F) and strongly accelerated the rate of vesicle recruitment (Fig 5I), leading to activity-dependent increases in total synchronous as well as asynchronous charge transfer (Fig 5G–5L). Thus, homomeric TRPC5 channels profoundly regulate synaptic plasticity. In contrast, TRPC1 expression in tko neurons failed to cause similar changes in STE-supporting synaptic responses that were indistinguishable from those in controls (Fig 5).

This observation verifies that the synaptic plasticity phenotype observed with lentiviral expression is not simply a consequence of potential off-target effects. It further suggests that the STE phenotype of TRPC1 expression in wt neurons (Fig 3) most likely relies on the interaction with the other TRPC variants. The latter notion agrees with observations in heterologous expression systems in which no evidence for functional homomeric TRPC1 channels was found [21,46,47]. To extend these findings, we transfected mass cultures of hippocampal wt and tko neurons with either TRPC1 or TRPC5 and recorded spontaneous miniature EPSC (mEPSC) events in the presence of tetrodotoxin (TTX; 10μM; blocking voltage-gated Na^+^ channels [48]). Expression of TRPC5 or TRPC1 increased the mEPSC frequency in wt cells without changing the kinetics of quantal events, consistent with a presynaptic function of these channels (S6 Fig). In the absence of other TRPC isoforms, only TRPC5, but not TRPC1, expression increased the mEPSC frequency (S6C and S6D Fig). Taken together, homomeric TRPC5 channels profoundly regulate synaptic plasticity and elevate the rate of spontaneous release. In contrast, TRPC1 differentially affects synaptic signaling in wt and tko neurons, most likely because it requires heteromultimerization with other members of its TRPC subgroup to form functional channels.

### Acute perturbation of TRPC activity interferes with synaptic signaling

To study whether acute inhibition of TRPC5 channels impairs synaptic efficacy, neurons were superfused with the TRPC5 inhibitor clemizole (3 μM, S7 Fig) [49]. In tko neurons expressing TRPC5, antagonist application significantly decreased STE of synaptic signaling and strongly reduced the asynchronous release when compared with the previous control response (S7B and S7C Fig). In contrast, clemizole neither affected synaptic depression nor asynchronous release in tko neurons, showing the specificity of the antagonist (S7D and S7E Fig). Collectively, the observed STE phenotype is due to immediate activation of TRPC channels during HFS, rather than being caused by developmental or compensatory mechanisms in response to TRPC channel expression.

**Fig 6:**
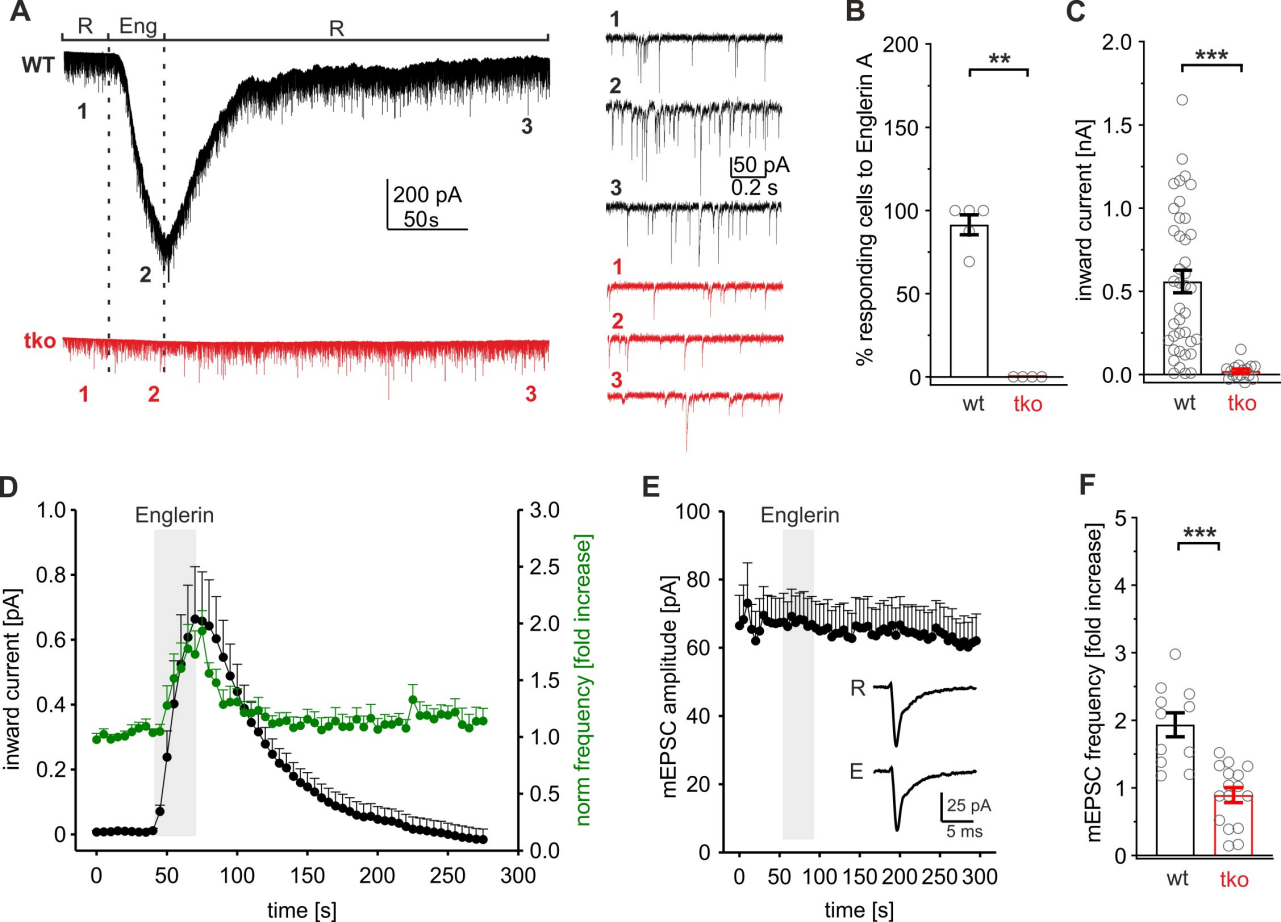
Englerin A activates presynaptic TRPC channels and increases the mEPSC frequency. (A) Exemplary recordings of autaptic wt and TRPC1/C4/C5-tko neurons superperfused with Ringer (R) solution containing Englerin A (Eng, 1 μM). Englerin A evoked an inward current in wt but not tko cells. Right panels, expanded timescale of the recording at the indicated time points. Note the clear mEPSC frequency increase in wt neurons with Englerin A application (2). (B) Percentage of cells responding to Englerin A with an inward current (*p* = 0.008). (C) Quantification of the maximum inward current amplitude (wt, *n* = 36; tko, *n* = 16; *p* = 4.3 × 10^−9^) determined at the end of Englerin A application relative to baseline current. (D) Time course of the averaged inward current (black) and the corresponding mEPSC frequency (green) during Englerin A application (40–70 s) in wt neurons (*n* = 11). (E) mEPSC amplitudes (determined for the cells shown in panel D) remain unchanged during Englerin A application. Insets depict averaged mEPSCs during Ringer (R, *n* = 72) and Englerin A (E, *n* = 65) application. (F) Englerin A evokes a 2-fold increase in mEPSC frequency (relative to the mEPSC frequency before drug application) in wt but not in tko neurons (wt, *n* = 36; tko, *n* = 16; *p* = 0.00004). **p < 0.01; ***p < 0.001, Mann-Whitney rank sum test. Underlying data can be found in S1 Data. mEPSC, miniature excitatory evoked postsynaptic current; tko, triple knockout; TRPC, transient receptor potential canonical; wt, wild type.

We next investigated whether direct activation of endogenous TRPC channels by the specific TRPC4/C5-agonist Englerin A [50] influences synaptic vesicle exocytosis. Autaptic wt neurons responded to agonist application in the presence of TTX (10 μM) with a reversible inward current (maximum current amplitude: 558 ± 68 pA, *n* = 36), whereas no significant current response could be detected in tko cells, verifying the specificity of Englerin A (Fig 6A, 6B and 6C). Interestingly, TRPC-mediated permeability changes were often paralleled by a transient increase in mEPSC frequency (Fig 6D, green line), without changing the amplitude or kinetics of the mEPSCs (Fig 6E). This suggests that activation of endogenous TRPC4/5 channels can directly evoke synaptic vesicle exocytosis. Some wt neurons (12 out of 36 cells) failed to respond to Englerin A (mEPSC frequency increase <1.2-fold), which could be due to heterogeneities in the expression or subcellular localization of TRPC4/C5 in hippocampal neurons. On average, Englerin A evoked a 2-fold increase in the mEPSC frequency of wt neurons (2.0 ± 0.28-fold increase over baseline levels, Fig 6F) but had no effect in tko neurons. To study the ionic basis of the inward current evoked by Englerin A, we repeated these experiments in neurons, which are genetically deficient for vesicular soluble N-ethylmaleimide-sensitive-factor attachment receptor (SNARE) proteins and are devoid of any glutamate release [48,51]. The results show that Englerin A evokes similarly large inward currents in the absence of any neurotransmitter release (S8 Fig). Thus, the observed inward current can be largely attributed to direct activation of TRPC channels rather than to secondary activation of glutamate receptors as a consequence of TRPC activity.

Collectively, these results are in line with an at least partial presynaptic localization of TRPC5 channels (S1 Fig) and compatible with the mEPSC frequency increase observed upon lentiviral TRPC expression (S6 Fig). They show that acute perturbation of TRPC activity is able to regulate STP and synaptic vesicle exocytosis.

### Enhanced Ca^2+^-entry through VGCCs does not mimic the TRPC phenotype

**Fig 7:**
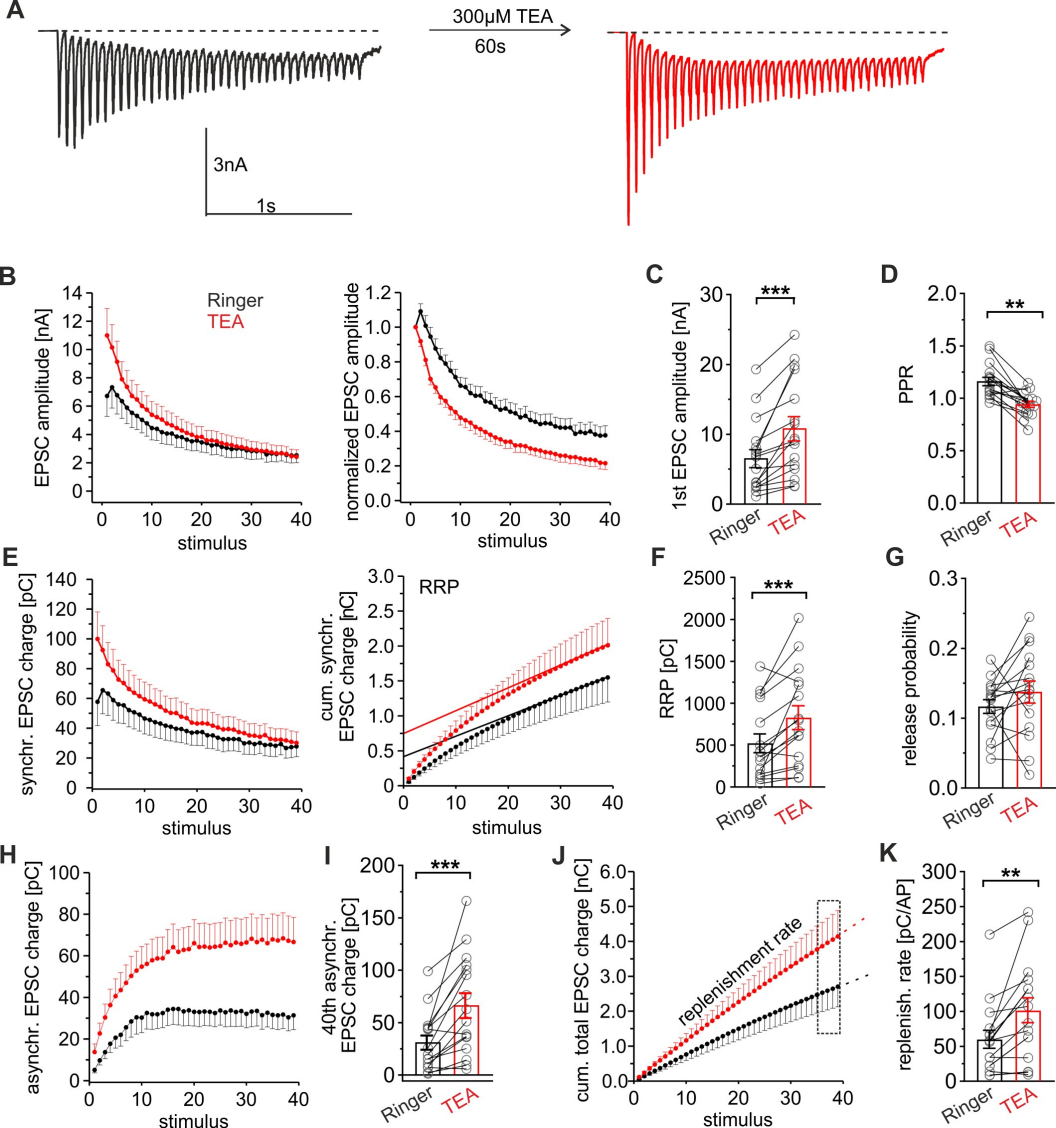
Elevated Ca^2+^ entry through VGCCs increases the EPSC amplitude and accelerates synaptic depression. (A) Representative EPSC recordings of a wt neuron (20 Hz, 40 AP/2 s) before and after TEA application (300 μM). (B) Time course of the EPSC amplitude for the first (Ringer) and the second train response (+TEA); right panel, data normalized to the first EPSC amplitude. Note that TEA increases the degree of STD. (C and D) TEA increases the initial EPSC amplitude (C) and decreases the PPR (D) (first amp: *p* = 0.00003; PPR: *p* = 0.0002). (E) Time course of the EPSC synchronous charge for Ringer and TEA (left) and its cumulative plot (right). Continuous line, linear regression of the last 5 data points to estimate the initial RRP size (shown in panel F). (F and G) The RRP size (F) and the Pr (G) are significantly larger with TEA (RRP: *p* = 0.0007; Pr: *p* = 0.24). (H) TEA increases asynchronous release. (I) Mean asynchronous release of the 40th EPSC (*p* = 0.0001). (J) Time course of the cumulative total synaptic charge transfer; dashed lines, linear regression of the last 4 data points to estimate the replenishment rate shown in panel K. (K) TEA elevates the replenishment rate (*p* = 0.003). Data was collected from 16 cells, *p < 0.05; **p < 0.01; ***p < 0.001; Student paired *t* test. Underlying data can be found in S1 Data. AP, action potential; EPSC, evoked postsynaptic current; PPR, paired-pulse ratio; Pr, release probability; RRP, readily releasable pool; STD, short-term depression; TEA, tetraethylammonium; VGCC, voltage-gated calcium channel; wt, wild type.

TRPC channels may either directly mediate Ca^2+^ influx into synaptic terminals or indirectly modulate synaptic plasticity through facilitated opening of voltage-gated Ca^2+^ channels. In the latter case, TRPC-channel–mediated depolarization of the membrane potential could ease VGCC opening and enhance Ca^2+^ entry into the presynaptic terminal. Broadening the presynaptic AP-width with the potassium channel blocker tetraethylammonium (TEA, 300μM) has been shown to enhance the VGCC-mediated Ca^2+^ influx and synaptic transmission during high-frequency stimulation [45]. Compared to the preceding control response, acute TEA application significantly increased the initial EPSC amplitude and decreased the PPR leading to an overall faster synaptic depression (Fig 7A–7D). These changes are paralleled by an increase in release probability (Fig 7G) and clearly contrast the TRPC phenotype. TEA also enhanced the synchronous release component and the RRP size. It furthermore augmented asynchronous secretion and increased the replenishment rate, consistent with a prolonged and stronger Ca^2+^ entry into synaptic terminals (Fig 7E–7K). Overall, the enhanced Ca^2+^ influx through VGCCs leads to stronger STD and clearly differs from the STE phenotype observed with higher TRPC activity. Thus, it is unlikely that synaptic TRPC channels merely increase the Ca^2+^ influx through depolarization-dependent modulation of VGCCs. The combined set of data suggests that TRPC channels provide an additional Ca^2+^ entry pathway most likely distal to the active zone, enabling efficient mobilization of dormant vesicles from the reserve pool and leading to STE of synaptic signaling during HFS.

### TRPC channels augment the presynaptic Ca^2+^ rise upon HFS

**Fig 8:**
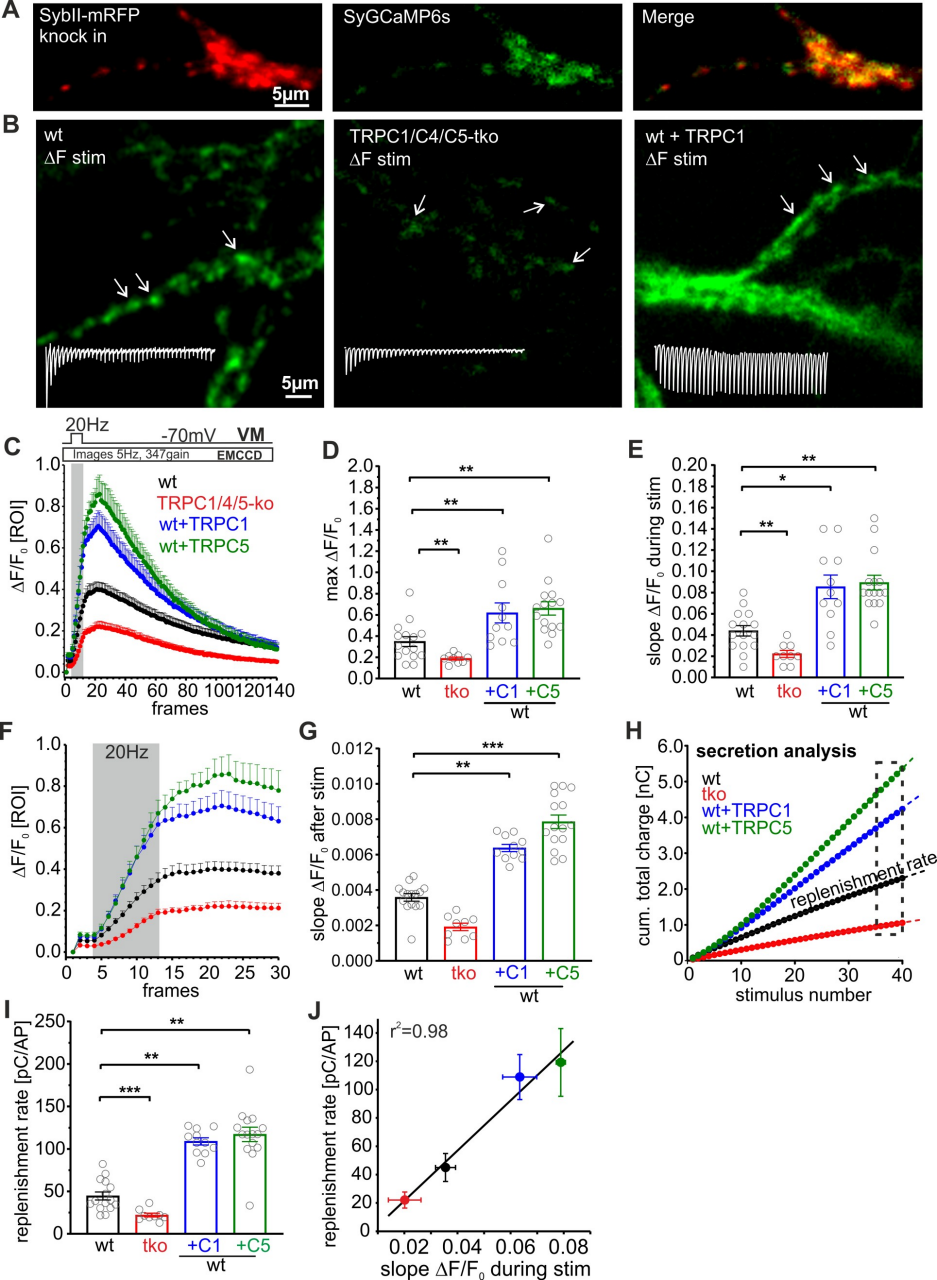
TRPC channels augment the presynaptic Ca^2+^-rise upon HFS. (A) Exemplary images of neurons expressing the vesicular protein SybII-mRFP (left) and Synaptophysin-GCaMP6s (SyGCaMP6s, middle). Note the high degree of colocalization between synaptobrevinII and SyGCaMP6s (right). (B) Sample difference images (ΔF/F_0_) of SyGCamp6s signaling recorded (5 Hz) from autaptic neurons (wt, left; tko, middle; wt + TRPC5, right) during the HFS (20 Hz, 2 s); insets are corresponding EPSC recordings from the same cell. Arrows are exemplary ROIs used to monitor ΔF/F_0_ at single synaptic sites. (C) Loss of TRPC channels decreases the presynaptic Ca^2+^ rise, whereas expression of either TRPC variant strongly increases the Ca^2+^ signal. (D) Maximum ΔF/F_0_ from the data shown in (C), tko, *p* = 0.006; +C1, *p* = 0.0076; +C5, *p* = 0.0023. (E) TRPC deficiency decreases, whereas TRPC expression increases the slope of ΔF/F_0_ during HFS (slope between the 6th and the 13th data point; tko, *p* = 0.003; +C1, *p* = 0.035; +C5, *p* = 0.002. (F) Expansion of the early phase of the plot shown in panel C illustrating the prolonged Ca^2+^ rise after HFS. (G) Expression of either TRPC variant significantly enhances the Ca^2+^ influx right after HFS (slope determined between the 14th and 20th data point; tko, *p* = 0.48; +C1, *p* = 0.025; +C5, *p* = 0.0003). (H) Corresponding mean cumulative total charge transfer of the neurons imaged in panel C. (I and J) The replenishment rate (determined from the slope of the cumulative plot, last 4 data points, shown in panel H, is significantly changed by altering TRPC expression (I) and correlates with changes in SyGCaMP6s (slope of ΔF/F0) during HFS); tko, *p* = 0.0002; +C1, *p* = 0.009; +C5, *p* = 0.0004. Data were collected from wt, *n* = 15; tko, *n* = 9; wt + TRPC1, *n* = 11; wt + TRPC5, *n* = 15; *p < 0.05; **p < 0.01; ***p < 0.001; one-way ANOVA on ranks followed by Dunn’s post hoc test versus wt. wt versus tko Mann-Whitney rank sum test. Underlying data can be found in S1 Data. AP, action potential; EMCCD, electron multiplying charge coupled device; EPSC, evoked postsynaptic current; HFS, high-frequency stimulation; mRFP, monomeric red fluorescent protein; ROI, region of interest; SybII, synaptobrevin II; SyGCaMP6s, Synaptophysin-GCaMP6s; tko, triple knockout; TRPC, transient receptor potential canonical; VM, membrane voltage; wt, wild type; ΔF, delta fluorescence.

To study how TRPC channels influence presynaptic Ca^2+^ dynamics, we combined electrophysiological recordings of synaptic activity with presynaptic Ca^2+^-imaging using the vesicle-associated Synaptophysin-GCaMP6s (SyGCaMP6s) fusion protein as a presynaptic Ca^2+^ reporter (Fig 8). In preparatory work, we verified that SyGCaMP6s is sorted to synaptic sites, as illustrated by its high degree of colocalization with the synaptic vesicle protein synaptobrevin II (SybII; Fig 8A). Autaptic neurons were stimulated with 20 Hz HFS for 2 s, while the synaptic charge transfer and [Ca]i changes at discrete synaptic sites were monitored simultaneously throughout the experiment. Discrete synaptic regions were analyzed when the fluorescence increase (ΔF/F0) exceeded 3 SDs of the background noise (ΔF/F0, mean ± SEM: 0.022 ± 0.00012). Wt neurons responded to electrical stimulation with a robust, activity-dependent increase in SyGCaMP fluorescence (Fig 8B and Fig 8C). Genetic loss of TRPC1/C4/C5 reduced the presynaptic Ca^2+^ rise, whereas the expression of either TRPC1 or TRPC5 strongly elevated presynaptic Ca^2+^ dynamics (Fig 8B, 8E and 8D). The slope of the fluorescence increase during HFS was nearly 2-fold higher in TRPC-expressing cells compared with wt neurons (Fig 8E), indicating that TRPC channels directly potentiate the VGCC-mediated increase in presynaptic Ca^2+^. Even after HFS, when VGCCs have closed, [Ca]i continued to increase more strongly in TRPC-expressing neurons than in wt and tko neurons (Fig 8F and 8G). These observations provide strong evidence that TRPC channels establish an additional Ca^2+^ entry pathway that functionally couples to the VGCC-mediated Ca^2+^-influx and is able to prolong the presynaptic Ca^2+^ signal. Importantly, alterations of the average vesicle replenishment rate determined from simultaneous electrophysiological recordings correlate well with the observed changes in presynaptic Ca^2+^ levels among the different groups (Fig 8I and 8J). Taken together, these observations demonstrate that TRPC channels augment and prolong the presynaptic Ca^2+^ increase upon HFS and, by this, set the pace of synaptic vesicle recruitment.

## Discussion

Changing the strength of synaptic connections between neurons is subject to different short-lived or long-lasting regulation processes, with the underlying cellular mechanisms often remaining enigmatic. In the present study, we employed a variety of genetic and lentiviral expression strategies to unravel the functional impact of Ca^2+^-permeable TRPC channels on synaptic efficacy of hippocampal neurons. Our experiments provide first evidence that systematic alterations in the expression of endogenous TRPC5 channels lead to graded changes in the synaptic plasticity behavior of glutamatergic synapses. Lentiviral expression of TRPC proteins in combination with presynaptic Ca^2+^-imaging unveiled a TRPC-dependent augmentation and prolongation of the intraterminal Ca^2+^ signal that accelerates the rate of vesicle replenishment and boosts STE of synaptic signaling. Overall, the results identify hitherto unknown TRPC-mediated mechanisms which profoundly alter synaptic plasticity at synapses in the central nervous system (CNS).

### Presynaptic TRPCs govern synaptic plasticity

Short-term synaptic plasticity regulates the strength of neurotransmission through facilitation and depression at the millisecond time scale and plays a key role in encoding information in the nervous system. VGCCs channels are the major source of Ca^2+^ entry for neurotransmission in the central nervous system. Yet, little is known about other Ca^2+^-entry channels, which may activate in response to Ca^2+^ influx through VGCCs and in turn further elevate the presynaptic Ca^2+^ rise. Such a scenario has been proposed for the activity-dependent activation of Ca^2+^-permeable transient receptor potential channels of the vanilloid subtype (TRPV1) receptors that promote asynchronous release from solitary tract afferents [52]. We have previously shown that [Ca]i elevations are essential and sufficient for the activation of TRPC5 [8]. TRPC5 channels are activated in a dose-dependent manner at submicromolar [Ca]i (effective concentration 50 [EC_50_] 635nM [8]) and are therefore well suited to sense changes in bulk [Ca]i. Furthermore, they respond to stepwise changes in [Ca]i at the millisecond time scale and thereby meet the speed requirements for regulating synaptic STP. Using independent experimental strategies, we show that TRPC channels reside at presynaptic sites and are able to regulate synaptic transmission. First, endogenous TRPC5 channels could be found in varicosity-like thickenings in which they colocalized with the presynaptic marker protein bassoon (S1 Fig). Second, genetic loss of TRPC1/C4/C5 reduced basal synaptic transmission, diminished the RRP size, and sped up the rate of synaptic depression during the HFS because of impeded vesicle recruitment (Fig 1). Third, acute activation of TRPC4/C5 channels by the agonist Englerin A stimulated an increase in mEPSC frequency, indicating that Ca^2+^ entry through TRPC channels is able to directly evoke small synaptic vesicle (SSV) exocytosis (Fig 6). Fourth, TRPC deficiency neither affected properties of quantal signaling, indicative of unchanged postsynaptic signal generation (S6 Fig), nor did it cause any changes in synapse number or organization (S4 Fig). Furthermore, by taking advantage of a TRPC5 channel reporter mouse strain (TRPC5-IC/eR26-τGFP), we avoided heterogeneities in channel expression and confined our measurements to neurons that express endogenous TRPC5 channels (Fig 2). These neurons exhibit strong STE of their synaptic response, enhanced asynchronous release, and an elevated replenishment rate. This behavior contrasts the classical synaptic depression during HFS usually observed in wt neurons and is characteristic for enhanced vesicle supply, leading to transient overfilling of the RRP during the stimulus train [53]. Moreover, loss of TRPC channels reduced presynaptic Ca^2+^ entry as documented by SyGCaMP6s measurements (Fig 8). Overall, these results show that endogenous TRPC channels crucially modulate presynaptic Ca^2+^ levels which in turn govern the rate of synaptic vesicle recruitment to release sites. These new observations also provide an attractive explanation for the functional deficits that underlie the impaired transient potentiation after 100 Hz stimulation in acute hippocampal slices of Trpc1/4/5^−/−^ tko mice [37].

Expression of either TRPC variant (C1 or C5) profoundly influenced STP and switched short-term depression into STE of synaptic signaling (Fig 3). The activity-dependent increase in synchronous and asynchronous release during HFS together with a higher PPR is remarkably similar to the phenotype of τGFP-positive neurons expressing endogenous TRPC5. This indicates that TRPC5 activity is crucial for changing the mode of synaptic plasticity and excludes the possibility that STP changes with lentiviral expression are simply due to an excess of these ion channels. Furthermore, it stands to reason that TRPC5 expression in tko neurons did not merely rescue the plasticity phenotype of wt neurons but instead induced substantial STE (compare Fig 5 and Fig 1). This result is most likely due to the absence of TRPC5 in a subpopulation of wt neurons (S2 Fig) and may also be the consequence of higher TRPC5 expression levels upon lentiviral transduction.

In tko neurons, expression of TRPC5 suffices to produce strong changes in STP, whereas TRPC1 neither changed evoked nor spontaneous release (Fig 5; S6 Fig). Thus, TRPC5 channels can function as homomeric channels to control presynaptic Ca^2+^ dynamics, but TRPC1 requires other members of its subfamily, as has been also observed in heterologous expression systems [20]. Given that isolated TRPC1 channels neither function as Ca^2+^-entry channels [54] nor are they directly activated by Ca^2+^ [27], the effects of TRPC1 expression in wt neurons may be explained by alterations in the stoichiometry of presynaptic TRPC channels or by changes in their subcellular distribution. Furthermore, other functionally relevant interactions of TRPC1 either with members of the store operated calcium entry (SOCE) family (Orai1, and stromal interaction molecule 1 [STIM1], [27]) or with those of a different TRPC subgroup (e.g., TRPC3/C6/C7 [54]) cannot be rigorously excluded. Nevertheless, our results demonstrate that loss and surplus of TRPC activity affect STP of hippocampal neurons in an opposite manner, either increasing or decreasing synaptic depression.

### A new model for the TRPC function in presynaptic Ca^2+^ homeostasis

Given that TRPC5 channels functionally couple to VGCCs in heterologous expression systems [8], we hypothesize that Ca^2+^ entry through VGCCs may trigger TRPC channel openings, which lead to additional Ca^2+^ influx and thereby modulate presynaptic Ca^2+^ homeostasis and synaptic plasticity. In good agreement, expression of either TRPC1 or C5 elevated the presynaptic Ca^2+^ rise right after the HFS, providing direct evidence that these ion channels are able to prolong the Ca^2+^ entry into presynaptic terminals (Fig 8). These observations are consistent with acute TRPC-mediated changes in presynaptic Ca^2+^ dynamics rather than overall alterations in presynaptic Ca^2+^ buffering. Moreover, the TRPC5 inhibitor clemizole acutely affects STE of synaptic signaling and asynchronous release, reinforcing the view that TRPC5 activity directly shapes presynaptic Ca^2+^ dynamics (S7 Fig).

Notably, the changes in presynaptic [Ca]i during HFS correlated well with alterations in the replenishment rates among the different groups (as observed with simultaneous electrophysiological recordings). In the same line, TRPC5 and C1 expression accelerated and strongly augmented the degree of recovery from synaptic depression, confirming that the TRPC-mediated Ca^2+^ permeability facilitates the refilling of the RRP through mobilization of synaptic vesicles from the reserve pool (S5 Fig).

In contrast to genetic TRPC deficiency, lentiviral transduction of TRPC channels in wt or tko neurons neither affected the first EPSC amplitude of the train response nor the RRP size (see Figs 1, 3, 5 and S3 Fig). It is possible that such changes are counterbalanced by high levels of spontaneous vesicle fusion as documented by the elevated mEPSC frequency of TRPC-expressing neurons (S6 Fig).

The comparative analyses between the TRPC1/C5 expression phenotype and the enhanced Ca^2+^ influx through VGCCs by TEA treatment revealed important functional differences (Fig 7). TEA-application enhanced asynchronous release and the vesicle replenishment rate but also increased the first EPSC amplitude, reduced the PPR, and showed a stronger synaptic depression during HFS. The latter results are characteristic of a higher release probability in the presence of TEA (see also [45,55]) and are most likely due to increased presynaptic [Ca]i in close proximity to the fusion site. Yet, they clearly differ from the TRPC expression phenotype, indicating that activation of distinct synaptic Ca^2+^ permeabilities differentially affects STP characteristics varying between STD and STE. An attractive explanation could be that VGCCs cluster at active zones [56], whereas TRPCs may reside at perisynaptic sites. In support of such a hypothesis, we found that preincubation with EGTA fully abolishes the TRPC effects on short-term plasticity (Fig 4). Assuming low millimolar concentrations of EGTA (approximately 1 mM) at the synaptic site, the Ca^2+^ buffer will chelate Ca^2+^ ions with a time constant of about 100 μs (τ_chelation_ [57]). Under these conditions and for a Ca^2+^ diffusion coefficient (D_Ca_) of 2.2 × 10^−6^ cm^2^sec^−1^ [58] Ca^2+^ can diffuse more than 350 nm (r^2^ = 6 × D_Ca_ × τ_chelation_) before efficient chelation by EGTA. These estimates are consistent with the idea that TRPC channels may reside at farther distances from the active zone, making them well positioned to enhance preferentially the Ca^2+^-dependent mobilization of reluctant vesicles from the reserve pool. Collectively, these experiments show that different types of synaptic plasticity result from enhanced Ca^2+^ influx through either VGCCs or TRPCs. They suggest that presynaptic Ca^2+^ homeostasis is a highly orchestrated process based on different compartmentalized Ca^2+^ entry pathways to ensure that efficient vesicle recruitment matches the high rates of vesicle fusion upon ongoing synaptic activity.

Alterations in TRPC channel expression may contribute to the known variability of synaptic strength and plasticity in hippocampal neurons [59]. Furthermore, the STP characteristics of enhanced TRPC activity are remarkably similar to those observed in mammalian uncoordinated 13–2 (Munc13-2) dependent synapses [60], including the frequency-dependent facilitation during HFS and the post-train augmentation (i.e., increased EPSC amplitudes after high-frequency train). Munc13-2 has been shown to facilitate basal synaptic vesicle (SV) priming, STE of synaptic signaling [61], and is recruited to synapses by the active zone protein glutamic acid/leucine/lysine/serine‐rich protein (ELKS1) [62]. Intriguingly, synaptic vesicle priming factor proteins like Munc13-2 or Double C2-protein (Doc2) [63], which sense Ca^2+^ either with their C2-domains or in a complex with calmodulin, are translocated to the plasma membrane in a Ca^2+^-dependent manner [64] and may serve as transducers of the TRPC-mediated effects on vesicle replenishment and short-term plasticity. Similarly, other EF-hand proteins such as the neuronal calcium sensor 1 (NCS1) protein may contribute to the TRPC phenotype. NCS1 is enriched in presynaptic terminals [65], interacts with TRPC channels [18], and has a strong impact on short-term plasticity of hippocampal neurons. Interestingly, overexpression of NCS1 in hippocampal neurons mimics the phenotype of STE [65] observed with TRPC5 expression, making it possible that both proteins are engaged in the same signaling pathway.

Taken together, our results identify TRPC channels as important modulators of presynaptic Ca^2+^ homeostasis and plasticity of fast glutamatergic synapses. They indicate that TRPC channels amplify the presynaptic Ca^2+^ rise and elevate the rate of synaptic vesicle recruitment to cope with vesicle consumption during high neuronal activity. Thus, TRPC-mediated changes in synaptic plasticity are well suited to play a central role for information processing in the central nervous system.

## Material and methods

### Cell culture and animals

TRPC1/C4/C5-tko mice were generated as described previously by Broker-Lai and colleagues [37]. Autaptic cultures of hippocampal neurons from age-matched TRPC tko, wt (C56BI/6N strain), and TRPC5-IC/eR26-τGFP mice were prepared from P0-1 animals as described previously by Schwarz and colleagues [66]. v-SNARE knockout animals (syb2^−/−^ or syb2^−/−^/ceb^−/−^-dko) and their littermate control were prepared at E18.5. Briefly, hippocampi were dissected from the brain and digested for 20 mins at 37°C with 10 units of papain (Worthington, NJ), followed by gentle mechanical trituration. Neurons were seeded at low density (1,000 cells/ml) onto a layer of glial microislands, resulting in co‐cultures of glia and neurons. For electrophysiological recordings, only islands with single neurons were used. For mass cultures, neurons were seeded with a density of 300 cells/mm^2^ on 25-mm cover slips coated with 0.5 mg/ml of poly‐D‐lysine (Sigma, Germany). Cultures were maintained at 37°C in an incubator, humidified with 95% air and 5% CO_2_ in NBA (Invitrogen), supplemented with 2% B‐27 (Sigma, Germany), 1% Glutamax (Invitrogen, Germany), and 1% penicillin/streptomycin (Invitrogen, Germany). Recordings were performed at room temperature on 9 to 13 days of culture.

### Construction of the TRPC5-IRES-Cre (TRPC5-IC) targeting vector

The final targeting construct includes a 5ʹ TRPC5 homology arm, an IRES-Cre-pgk promoter-driven Flp recombination target (FRT)–neomycin (neo)–FRT cassette and a 3ʹ TRPC5 homology arm. Using genomic R1 mouse embryonic stem (ES) cell DNA, the 2587-bp 3ʹ homology arm containing sequence downstream of the final exon of TRPC5 (exon 11) was amplified by polymerase chain reaction (PCR). By incorporation of restriction enzyme sequences within the primers, an AscI site was added 5ʹ to the homology arm and a BamHI site added at the 3ʹ end, and the fragment was subcloned into pKO-DTA. Using a similar strategy, the 2417-bp 5ʹ homology arm containing the stop codon of TRPC5 was generated and also cloned into the vector using XhoI and AscI (New England Biolabs, Germany) sites. PCR amplification of both homology arms was undertaken using the high-fidelity pfu DNA polymerase to minimize PCR-induced mutations, and any nucleotides that differed from the database sequence upon sequence analysis were verified by independent PCR amplification and sequencing. In a final step, the IRES-Cre-pgk promoter-driven FRT-neo-FRT cassette was cloned into the AscI (New England Biolabs, Germany) site found at the junction of the 2 homology arms. The completed targeting construct was then further verified by a complete sequence analysis and restriction mapping.

### Gene targeting

Following verification of the integrity of the targeting construct, plasmid DNA was linearized using the NotI enzyme and then electroporated into R1 ES cells at the GIGA institute, University of Liege. Following electroporation, Southern blot analysis was utilized to identify correctly targeted clones, and these were then used to generate mice following standard protocols (injection of ES cells [129/Sv] into blastocysts [C57BL/6], implantation of injected blastocysts into foster mothers, backcross of male chimeras with C57BL6 females). F1 animals resulting from backcrosses were then crossed with FLP-deleter mice, which contain a ubiquitously expressed FLP recombinase gene, to facilitate removal of the neomycin selection cassette.

### Viral contructs/transfections

The cDNAs encoding for TRPC1, TRPC5 (NCBI accession numbers NM_011643 and NM_009428.2), and SyGcamp6s (Addgene #26124) were subcloned into the plenti-hsynapsin lentiviral transfer vector. mRFP was fused to the C-terminal end of TRPC5 with a flexible 12 amino acid linker sequence. All constructs were verified by DNA sequence analysis (MWG Germany). Lentiviral particles were produced as previously described by Schwarz and colleagues [66]. Primary neurons were transfected with 100 to 300 μL of viral suspension 1-3DIC.

### Drug treatment

All chemicals were purchased from Sigma-Aldrich unless stated otherwise. For Englerin A (Roth, Germany 0.2–1 μM), TEA, clemizole (Tocris, United Kingdom), and sucrose experiments, neurons were rapidly superfused using a gravity-fed fast-flow system. For EGTA-AM pretreatment, neurons were incubated for 5 min in nominally Ca^2+^ free Ringer’s solution in the presence of 300 μ M EGTA-AM (Merck, Germany) and subsequently washed with Ca^2+^-containing Ringer’s solution before recording.

### Electrophysiological measurements of synaptic currents

Synaptic currents were recorded in the whole-cell voltage clamp mode from autaptic neurons. Patch pipettes (R_tip_ 4–5.5 MOhm) were filled with the following intracellular solution (in mM): 137.5 K-gluconate, 11 NaCl, 2 MgATP, 0.2 Na_2_GTP, 1.1 EGTA, 11 HEPES, and 11 D-glucose (pH 7.3) with KOH. The standard extracellular solution containing (in mM) 130 NaCl, 10 NaHCO_3_, 2.4 KCl, 1 to 2 CaCl_2_, 2 MgCl_2_, 10 HEPES, and 10 D-glucose (pH 7.3) with NaOH, osmolarity 299 mOsm was used. D-2-amino-5-phosphonopentanoate (APV; 50 μM) was added to inhibit NMDA-receptors and prevent synaptic plasticity changes. The reversal potential of chloride-mediated currents was adjusted to the holding potential to avoid the potential contribution of GABAergic currents. Exemplary neurons were treated with 25 μ M DNQX (Sigma, Germany) to confirm the recordings of AMPA receptor (AMPAR) currents. Neurons were voltage-clamped at -70 mV with an EPC10 amplifier (HEKA Electronic, Germany) under control of Pulse 8.5 program (HEKA Electronic, Germany) and stimulated by membrane depolarizations to +10 mV for 0.7 ms every 5 s (0.2 Hz). Cells with an average access resistance of 6 to 15 mOhm, with 75% to 80% resistance compensation and <100 pA leak-current were analyzed. Current signals were low-pass filtered at 2.9 kHz (4 pole Bessel filter EPSC10) and digitized at a rate of 10 or 50 kHz. Spontaneous mEPSCs were recorded prior to stimulation. mEPSC recordings in mass neuronal cultures were performed in the presence of TTX (10μM). To determine mEPSC properties with reasonable fidelity and to prevent the detection of “false events” (due to random noise fluctuations), spontaneous mEPSCs with a peak amplitude exceeding >5 times the standard deviation of the baseline noise and a charge criterion >25 fC were analyzed using a commercial software (Mini Analysis, Synaptosoft, Version 6.0.3). The AP-evoked EPSC amplitude and charge were determined from the average of 10 EPSCs recorded at 0.2 Hz.

In hypertonic sucrose experiments, neurons were rapidly superfused with sucrose solution (500 mM sucrose, 5 s) using a gravity-fed fast-flow system. The RRP size was quantified from the charge integral of the current signal in response to sucrose application after subtracting the steady-state current component (determined at the end of hypertonic sucrose application), which probably reflects steady-state vesicle replenishment and exocytosis during hypertonic challenges [42].

The RRP size during HFS was quantified from the cumulative synchronous EPSC charge integral during the 20 Hz train. For this, the total charge of each AP evoked response within the train was corrected by subtracting the integral of the steady-state current component (= asynchronous charge) determined at the end of the stimulus interval [66]. The cumulative plot of the resulting synchronous release component reports the decrement in RRP size followed by a sustained phase of charge increase reflecting the steady-state phase of ongoing RRP vesicle replenishment. The sustained phase of secretion (last 5 data points) was approximated by linear regression. When back-extrapolated to time 0, its y-intercept provides an estimate of the RRP size with minimal contribution of refilling (see Fig 1L).

### Imaging SyGCaMP6s responses

SyGCaMP6s fluorescence was acquired with an Evolve EMCCD camera (Visitron, Germany) using a Zeiss Plan Apochromat 40× oil immersion objective (NA 1.3) on a Axiovert200 microscope (Zeiss). Autaptic neurons were stimulated with 20 Hz for 2 s. Fluorescent images were captured at 5 Hz with custom written macros in VisiView (Visitron, Germany), processed offline using ImageJ 1.43 software and SigmaPlot 13. The background subtraction was done by subtracting the F_0_ image (average of 3 prestimulus images) from all subsequent images (ΔF_n_ = F_n_ − F_0_). Regions of interest of identical size (4 × 4 pixels) were placed over single synapses reacting to electrical stimulation, and fluorescence changes were tracked throughout the stack.

### Immuncytochemistry

Neurons were fixed for 10 min (RT) in PBS containing 4% paraformaldehyde. Cells were quenched for 10 min with 50mM NH_4_Cl in PBS, blocked for 30 min in PBS containing 3% BSA and 0.1% TritonX100. Primary (anti-TRPC5, 1:100, affinity-purified rabbit polyclonal home-made; anti-bassoon, 1:500, mouse monoclonal, Synaptic Systems; anti-GFP, 1:500, guinea pig; Synaptic Systems, anti-PSD95, 1:500, rabbit polyclonal, Synaptic Systems, Germany) and secondary antibodies (1:1,000, Alexa-Fluor 555, Alexa Fluor 488 and Alexa Fluor 643-conjugated goat anti-mouse or goat anti-rabbit; Invitrogen, Germany) were diluted in blocking buffer. The anti-TRPC5 antibody was generated by immunization of rabbits with a C-terminal protein fragment (amino acids 766–969) of mouse TRPC5 (NCBI accession number NM_009428.2). Cells were incubated with primary and secondary antibodies overnight and for 1.5 h at RT, respectively. In some experiments, cells were treated with DAPI (200 nM; Invitrogen, Germany) for 5 min at RT before mounting in glycerol cells were imaged either on a confocal microscope (LSM 710; Zeiss, Germany) using AxioVision 2008 software (Zeiss, Germany) or an Axiovert200 (Zeiss, Germany), fluorescence was elicited with a Polychrome V monochromator (Till Photonics, Germany) and captured with a EMCCD camera (Evolve, Visitron, Germany). The following objectives and filter sets were used: 100×, 1.3 NA; 63×, 1.4 NA; 40×, 1.3 NA; 25×, 0.8 NA oil objectives, Zeiss 10 (BP 450–490; FT 510, BP 515–565), Zeiss 09 (BP 450–490, FT 510, LP 515), Zeiss 15 (BP 546/12, FT 580, LP 590), Zeiss 38 (BP 470/40, FT 495, LP BP 525/50), respectively.

### Image analysis

Images were analyzed with the software package ImageJ (version 1.45), AxioVision 2008 software (Zeiss, Germany), and SigmaPlot 13.0 (Systat Software, Inc.). For confocal imaging, optical sectioning was achieved with a 1 airy unit pinhole setting. In case of z-stack acquisition, horizontal image planes were separated by 0.480 μm in the stack. The acquisition settings were optimized to avoid underexposure and oversaturation effects and kept equal throughout image acquisition of control versus knockout samples. Thresholding and background corrections were performed with identical settings for a given set of images acquired from control versus knockout samples. Presynaptic boutons were identified in single planes of the anti-bassoon image stacks in fine axonal structures to avoid contaminations with somatic TRPC5 fluorescence. Identified regions of interest (ROIs) were stored and used to quantify the Mander´s colocalization coefficient between bassoon and TRPC5. For confocal imaging, the percentage of TRPC5 and τGFP immunopositive cells was quantified relative to the number of nuclei profiles visible in the corresponding 40× bright field image. The number of TRPC5 positive cells was quantified by counting the number of DAPI positive nuclei that showed somatic TRPC5 staining. The number of synapses was determined after the images were subjected to uniform background subtraction (23 ± 2.1 au). Identical ROIs (3 × 3 pixels) were placed around bassoon-positive puncta on the entire autaptic neuron cell surface area throughout the stack (15–28 sections). Presynaptic bassoon-positive puncta were counted manually. To examine the spatial relationship between pre- and postsynaptic marker proteins, images were thresholded with 3× SD of the background fluorescence for PSD-95 and with a uniform threshold of 75 au for bassoon. Bassoon-positive puncta were detected using the Analyze Particle function in ImageJ. Enlarged ROIs (up to a 770 nm distance from the bassoon signal = “bassoon area”) were superimposed onto the PSD95 channel to determine the degree of colocalization and/or juxtaposition of both immunosignals within the ‘bassoon area’.

### Statistical analysis

Values are given as means ± SEM. To determine statistically significant differences, one-way ANOVA and a Student *t* test for comparing groups were used, if not indicated otherwise. The similarity of variances between groups was tested when performing statistics in SigmaPlot, and statistical tests were chosen accordingly. Normality was tested (Shapiro-Wilk). Multiple comparisons were performed using the Tukey-Kramer post hoc test. No statistical methods were used to predetermine sample sizes; however, sample sizes were similar to those employed in the field. Data collection and analyses were not performed blind to the experimental condition; no method of randomization was done.

### Online supplemental material

S1 Fig shows the subcellular distribution of TRPC5 channels in hippocampal neurons. S2 Fig shows the genetic targeting strategy used to express Cre recombinase under control of the TRPC5 promoter and the heterogeneous expression of TRPC5 in hippocampal neurons. S3 Fig shows that TRPC channels do not affect the basal release probability. S4 Fig shows that neither loss nor expression of TRPC channels affects the synapse number. S5 Fig shows that increased TRPC activity increases the rate of recovery from short-term depression. S6 Fig shows that increased TRPC5 activity elevates the mEPSC frequency. S7 Fig shows that the TRPC antagonist clemizole reduces STE of synaptic signaling. S8 Fig shows the Englerin A evoked inward current reports direct activation of TRPC5 channels.

## Supporting information

S1 Fig TRPC5 is localized to presynaptic terminals. (A) Co-immunolabeling of wt and TRPC1/C4/C5-tko neurons with the presynaptic marker protein bassoon (left panels) and TRPC5 (middle). Endogenous TRPC5 is found in somatic, dendritic, and axonal regions. It colocalizes in varicosity-like thickenings of soma-proximal and soma-distal fine axonal branches with the presynaptic marker protein bassoon (middle panels, magnified view of the boxed area shown in panel A. (B) No discernable immunosignal for TRPC5 could be detected in TRPC1/C4/C5-tko neurons. (C and D) Line-scan analyses (dashed lines shown in panels A and B) confirm the high degree of colocalization between TRPC5 (red) and bassoon (green). (E) TRPC5 colocalizes with bassoon in presynaptic terminals in wt cells. No TRPC5 signal could be detected in tko neurons. The colocalization was determined in thin axonal structures to avoid the contribution of somatic or dendritic TRPC5 staining. Data were collected from wt neurons (19 images, 179 ROIs) and tko neurons (21 images, 168 ROIs) prepared from 3 independent cultures. ***p = 1.5 × 10^−11^, Mann-Whitney rank sum test. Underlying data can be found in S1 Data. ROI, region of interest; tko, triple knockout; TRPC, transient receptor potential canonical; wt, wild type.
(TIF)

S2 Fig Heterogeneous expression of TRPC5 in hippocampal neurons. (A) Generation of the TRPC5-IRES-Cre mouse strain. Schematic representation of the targeting strategy used to express Cre recombinase under control of the TRPC5 promoter. From top to bottom, the targeting vector, the *TRPC5* wt allele and the targeted *TRPC5* allele before (neo^+^) and after (neo^−^) removal of the neomycin cassette are shown. The restriction sites for *Hpa*I and the location of the probe are indicated. The inserted cassette is composed of an IRES followed by the coding sequence for Cre recombinase (Cre) and a *pgk* promoter-driven neomycin selection cassette flanked by FRT sites. (B) Genotyping of TRPC5-IRES-Cre mice. Representative genotyping result from mice already carrying the neoallele, expected product sizes are 364 bp for wt and 500 bp for neoallele. Lanes 4, 5, and 6 represent TRPC5-IRES-Cre neohomozygous, heterozygous, and wt mice, respectively. Sites of primer annealing are shown in panel A; primers 1 and 2 in combination produce the wt band, and primers 3 and 2 produce the TRPC5-IRES-Cre neoband. (C) Confocal images of τGFP immunoreactivity (left panel) and TRPC5 staining (right panel) in hippocampal neurons (12 div) prepared from TRPC5-IC/eR26-τGFP mice. All τGFP-positive neurons (arrows) express TRPC5. Some cells positive for TRPC5 by immunostaining do not show τGFP staining (asterisk), indicating that τGFP-positive neurons represent a subset of TRPC5 expressing cells. (D) τGFP labeling faithfully reports TRPC5 expression, yet 38.5% ± 3.58% of the τGFP-negative cells were found to TRPC5 positive (data were collected from 831 cells, 2 preparations). No discernable immunosignal for TRPC5 was detected in tko neurons (*n* = 101 cells, 2 preparations). (E) Hippocampal wt neurons stained with DAPI (left panel, overlaid epifluorescence + brightfield channel) and immunolabeled with TRPC5 antibody (right panel). Only a subpopulation of hippocampal neurons (52% ± 3%, 195 out of 375 cells, 3 preparations) is immunopositive for TRPC5. No discernable staining could be detected within tko neurons, supporting the specificity of antibody reaction (lower panels). (F) Quantification of TRPC5 expressing cells as percentage of DAPI-stained cells. Underlying data can be found in S1 Data. Cre, cre-recombinase; DAPI, 4′,6-Diamidine-2′-phenylindole dihydrochloride; eR26, ROSA26-floxed-stop; FRT, Flp recombination target; IC, internal ribosomal entry site cre recombinase; IRES, internal ribosome entry site; neo, neomycin; tko, triple knockout; TRPC, transient receptor potential canonical; wt, wild type; τGFP, τ-green fluorescent protein.
(TIF)

S3 Fig TRPC channels do not affect the basal release probability. (A) Representative traces of the averaged evoked response (left panel) and the secretory response to 5 s application of 500 mM hypertonic sucrose solution (left panel) for wt, tko, wt + TRPC1, and wt + TRPC5 cells. (B) The evoked EPSC amplitude (left) and EPSC charge (right) are significantly reduced in tko neurons (amp: wt versus tko, *p* = 0.026; wt versus +C1, *p* = 0.31; wt versus +C5, *p* = 1.0; charge: wt versus tko, *p* = 0.01; wt versus +C1, *p* = 0.11; wt versus +C5, *p* = 0.9). (C) The RRP charge (left panel), determined by the time integral over hypertonic response, is reduced in tko cells (wt versus tko, *p* = 0.0004; wt versus +C1, *p* = 0.9; wt versus +C5, *p* = 0.9). The release probability determined by the ratio of EPSCcharge/RRPcharge is unchanged (right panel; wt versus tko, *p* = 0.9; wt versus +C1, *p* = 0.2; wt versus +C5, *p* = 0.9). Data were collected from wt, *n* = 53; tko, *n* = 42; wt + TRPC1, *n* = 27; wt + TRPC5, *n* = 26; *p < 0.05; one-way ANOVA on ranks followed by Dunn’s post hoc test. Underlying data can be found in S1 Data. EPSC, evoked postsynaptic current; RRP, readily releasable pool; tko, triple knockout; TRPC, transient receptor potential canonical; wt, wild type.
(TIF)

S4 Fig Neither loss nor expression of TRPC channels changes the synapse number or organization. (A) Exemplary confocal images (displayed as maximum intensity projections over 15 sections) of autaptic wt, tko, and wt neurons expressing TRPC1 or TRPC5. Immunolabeling with the presynaptic marker protein bassoon revealed no differences in the number of synapses. (Right panels) Higher magnification of presynaptic terminals from a single confocal section from the corresponding image on the left(dashed box). (B) The number of synapses (identified by counting single bassoon-positive puncta throughout the stack) was unchanged among the different groups (one-way ANOVA on ranks followed by Dunn’s post hoc test, *p* = 0.742). Data were collected from wt, *n* = 19; tko, *n* = 20; wt + TRPC1, *n* = 18; wt + TRPC5, *n* = 18. (C) Exemplary confocal image of cultured hippocampal wt neurons immunostained for the active zone protein bassoon and the postsynaptic density protein PSD-95. Bassoon and PSD-95 partially colocalize but more often occupy adjacent domains, consistent with their localization in pre- and postsynaptic compartments. Solid lines define the area wherein the colocalization or apposition of bassoon and PSD95 was quantified. Images are displayed as maximum intensity projection of 3 z-planes. (D) Neither loss of TRPC1/C4/C5 (tko) nor expression of TRPC1 or C5 in wt neurons affects the apposition of bassoon and PSD-95 when compared with controls. Values are given as mean of median determined from the parameter’s frequency distribution for each cell. Data were collected from wt, *n* = 16; tko, *n* = 14; wt + TRPC1, *n* = 12; wt + TRPC5, *n* = 12; *p* = 0.985; one-way ANOVA on ranks followed by Dunn’s post hoc test. (E) Mean frequency distributions of the relative “bassoon area” covered by PSD-95 staining for wt, wt neurons expressing TRPC1 or TRPC5, and tko neurons. Statistical power (pr) was determined with a post hoc power analysis. Underlying data can be found in S1 Data. PSD-95, post synaptic density protein 95; tko, triple knockout; TRPC, transient receptor potential canonical; wt, wild type.
(TIF)

S5 Fig Elevated TRPC activity increases the rate of recovery from short-term depression and leads to post-train augmentation of the synaptic response. (A) Representative EPSC traces (20 Hz, 2 s) of wt (black) and TRPC5-expressing wt neurons (green). Following HFS, the time course of recovery was determined by test pulses given after 20, 50, 150, 350, 650, 1,150, 2,150, and 4,150 ms intervals. (B) Time course for the recovery of the EPSC amplitude. Fraction of recovery was determined by dividing EPSC amplitudes of the test pulses by the first amplitude of the HF train. Data were fitted with a single exponential function (EPSC_(t)_ = k_off_ + EPSC_(∞)_ × (1-exp^-(t/τ)^), revealing reduced depression during HFS (k_off_, C), faster recovery kinetics (τ, D), and transient post-train augmentation of the synaptic response (EPSC_(4s)_, E) with expression of TRPC1 or TRPC5. (Inset) expansion of the early phase of the plot after normalization to the wt response. (C) TRPC1 or C5 expression increases k_off_ because of diminished synaptic depression during HFS (+C5, *p = 0.025; +C1, *p = 0.018; versus wt). (D and E) TRPC1 or TRPC5 expression significantly speeds up the recovery from depression (D; wt versus +C5, *p = 0.039, wt versus +C1, *p = 0.049; one-way ANOVA on ranks followed by Dunn’s post hoc test) and leads to post-train augmentation of the synaptic response (E; +C5, *p* = 0.0421, wt versus +C1, *p* = 0.0009, versus wt). Data were collected from wt, *n* = 29, wt + TRPC1, *n* = 21, wt + TRPC5, *n* = 16. (F) tko neurons show a slower rate of recovery from depression. Analysis was restricted to cells with significant depression during HFS to determine the time course of recovery with reasonable fidelity (wt, *n* = 44; tko, *n* = 25 cells; ***p = 0.000003; Mann-Whitney rank sum test). (G) The post-train augmentation of the EPSC was not significantly altered for tko neurons. (H) Time course of recovery for wt and tko cells (*p* = 0.73, Mann-Whitney rank sum test). Loss of TRPC channels slows down the rate of recovery from HFS; data were collected from wt, *n* = 57; tko, *n* = 25 cells. Underlying data can be found in S1 Data. EPSC, evoked postsynaptic current; HF, high frequency; HFS, high-frequency stimulation; tko, triple knockout; TRPC, transient receptor potential canonical; wt, wild type
(TIF)

S6 Fig TRPC5 expression suffices to elevate spontaneous glutamatergic signaling in tko neurons. (A) Exemplary recordings of spontaneous mEPSC signaling in neuronal mass cultures recorded in the presence of 10 μ M TTX. (B) Expression of either TRPC1 or TRPC5 significantly increases the mEPSC frequency (left panel). Corresponding cumulative frequency distributions of the mEPSC interevent interval (right panel). Data were collected from wt, *n* = 25; wt + C1, *n* = 22; *p* = 0.006; wt + C5, *n* = 20; *p* = 0.0001 cells. (C) The kinetics of the quantal events as illustrated for the indicated parameters remained unaffected by TRPC expression. (D) Sample recordings of mEPSC signaling from tko neurons expressing either TRPC1 or TRPC5. (E) Expression of TRPC5 but not of TRPC1 promotes an increase in mEPSC frequency (left panel). Corresponding cumulative frequency distributions of the mEPSC interevent interval (right panel). (F) mEPSC signaling with respect to amplitude and kinetics remained unchanged. Data were collected from tko, *n* = 20; tko + C1, *n* = 18; *p* = 0.91; tko + C5, *n* = 20; *p* = 0.002. **p < 0.01, ***p < 0.001; one-way ANOVA on ranks followed by Dunn’s post hoc test. Underlying data can be found in S1 Data. mEPSC, miniature evoked excitatory postsynaptic current; tko, triple knockout; TRPC, transient receptor potential canonical; TTX, tetrodotoxin; wt, wild type.
(TIF)

S7 Fig TRPC5 antagonist clemizole reduces STE of synaptic signaling. (A) Sample EPSC recordings of TRPC5-expressing tko neurons during 20 Hz HFS before (left) and after application of clemizole (3 μM, 90 s; right). (B) Time course of the EPSC amplitudes during HFS for Ringer and subsequent clemizole application. (Right panel) Clemizole significantly diminishes the EPSC amplitude (10th pulse; *p* = 0.016). (C) The asynchronous charge (determined for the 40th AP during the train) is significantly decreased with clemizole superfusion (*p* = 0.031). (D and E) In tko neurons, clemizole application neither affects the EPSC amplitude (10th pulse) nor the asynchronous charge (E). Data were collected from 3 independent preparations, tko, *n* = 16; tko + TRPC5, *n* = 17; *p < 0.05; paired *t* test. Underlying data can be found in S1 Data. AP, action potential; EPSC, evoked postsynaptic current; STE, short-term enhancement; tko, triple knockout; TRPC, transient receptor potential canonical.
(TIF)

S8 Fig The EA evoked inward current reports direct activation of TRPC5 channels. (A) Exemplary recordings of autaptic wt and synaptobrevin2-deficient neurons (syb2^-/-^) superfused with Ringer (R) solution containing EA (1 μM). No mEPSCs could be recorded in syb2^−/−^ neurons indicative of the exocytotic block upon genetic loss of syb2. (B and C) Neither the time course (B) nor the peak amplitude (C) of the EA-evoked current response was significantly altered in neurons lacking syb2 or in the common absence of syb2 and its homolog cellubrevin (syb2^−/−^/ceb^−/−^, dko). Data were pooled from syb2 ko and dko animals because loss of syb2 is crucial for abolishing transmitter release, and no significant differences between the phenotype of the corresponding littermate controls (wt neurons and ceb ko neurons) were detected. Data were collected from littermates (*n* = 13) and syb^−/−^ or syb^−/−^/ceb^−/−^ (*n* = 16); *p* = 0.983; Mann-Whitney rank sum test. Underlying data can be found in S1 Data. ceb, cellubrevin; dko, synaptobrevin2 and cellubrevin double knock out; EA, Englerin A; ko, knock out; mEPSC, miniature evoked excitatory postsynaptic current; syb2, synaptobrevin2; TRPC, transient receptor potential canonical; wt, wild type.
(TIF)

S1 Data Data underlying Figs 1–8 and S1–S8 Figs. (XLSX)

## Abbreviations

Amp: amplitude
AMPAR: AMPA receptor
AP: action potential
APV: D-2-amino-5-phosphonopentanoate
Ca]i: intracellular calcium concentration
CNS: central nervous system
Cre: Cre-recombinase
DAPI: 4′,6-Diamidine-2′-phenylindole dihydrochloride
Doc2: Double C2-protein
EA: Engerlin A
EC_50_: effective concentration 50
EGTA-AM: ethylene glycol-bis(β-aminoethyl ether)-N,N,N′,N′-tetraacetic acid acetoxymethyl ester
ELKS1: glutamic acid/leucine/lysine/serine‐rich protein
EMCCD: electron multiplying charge coupled device
EPSC: evoked postsynaptic current
eR26: ROSA26-floxed-stop
ES: embryonic stem
FRT: Flp recombination target
HFS: high-frequency stimulation
IC: internal ribosomal entry site cre recombinase
IRES: internal ribosome entry site
KI: knockin
mEPSC: miniature excitatory postsynaptic current, mRFP, monomeric red fluorescent protein
Munc13-2: mammalian uncoordinated 13–2
NCS1: neuronal calcium sensor 1
neo: neomycin
PCR: polymerase chain reaction
PPR: paired-pulse ratio
Pr: release probability
PSD-95: post-synaptic density protein 95
ROI: region of interest
RRP: readily releasable pool
SNARE: soluble N-ethylmaleimide-sensitive-factor attachment receptor
SOCE: store operated calcium entry
SSV: small synaptic vesicle
STD: short-term depression
STE: short-term enhancement
STIM1: stromal interaction molecule 1
STP: short-term plasticity
SV: synaptic vesicle
SybII: synaptobrevin II
SyGCaMP6s: Synaptophysin-GCaMP6s
TEA: tetraethylammonium
tko: triple knockout
TRP: transient receptor potential
TRPC: transient receptor potential canonical
TRPV1: transient receptor potential channels of the vanilloid subtype
TTX: tetrodotoxin
VGCC: voltage-gated calcium channel
VM: membrane voltage
wt: wild type
ΔF: delta fluorescence
τGFP: τ-green fluorescent protein
