# Supplementary figures and images for: TRPC channels regulate Ca2+-signaling and short-term plasticity of fast glutamatergic synapses
Source: PLoS Biol. 2019 Sep 19;17(9):e3000445. doi: 10.1371/journal.pbio.3000445 (PMC6773422; doi:10.1371/journal.pbio.3000445)

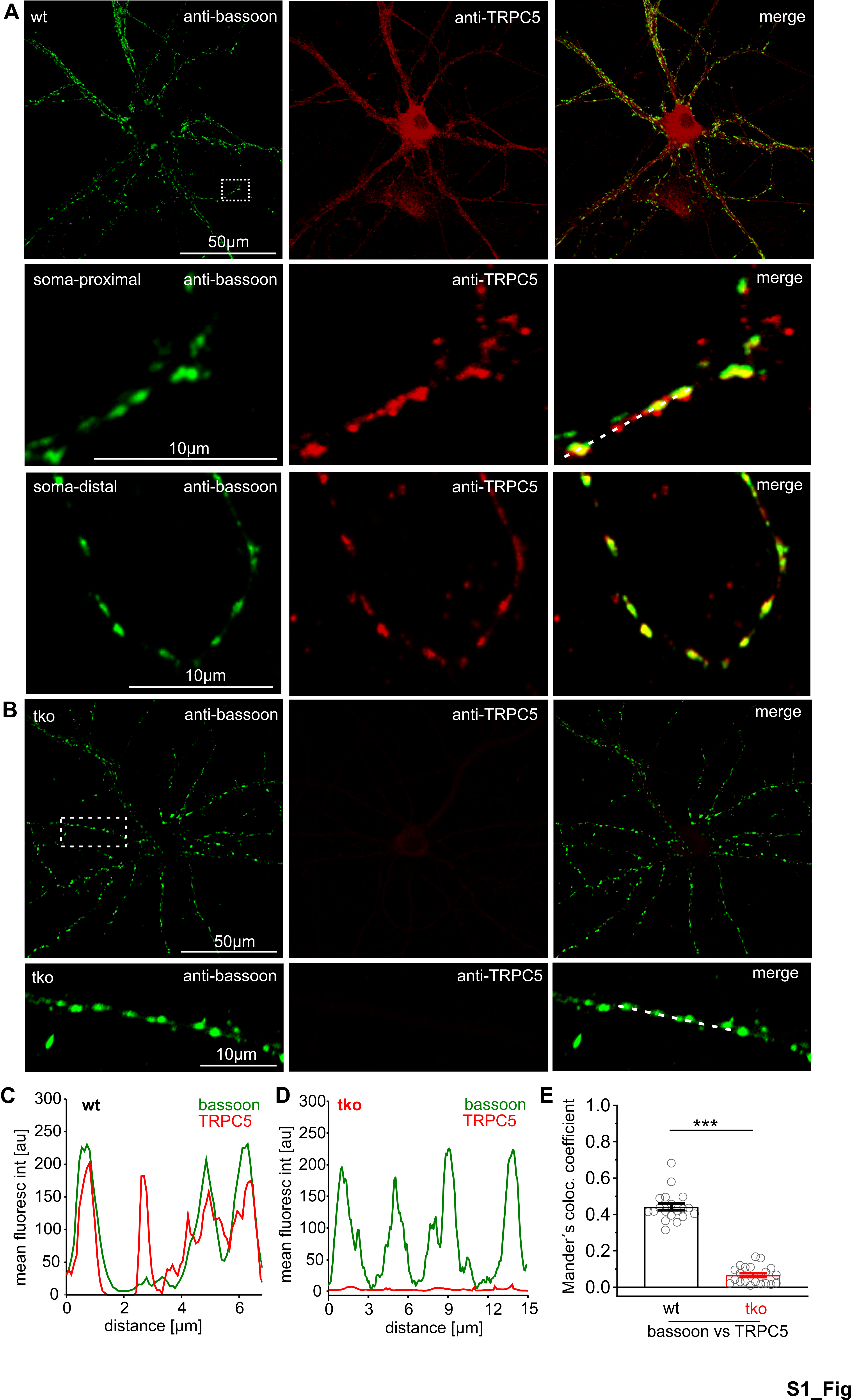

Supplement: S1 Fig — (A) Co-immunolabeling of wt and TRPC1/C4/C5-tko neurons with the presynaptic marker protein bassoon (left panels) and TRPC5 (middle). Endogenous TRPC5 is found in somatic, dendritic, and axonal regions. It colocalizes in varicosity-like thickenings of soma-proximal and soma-distal fine axonal branches with the presynaptic marker protein bassoon (middle panels, magnified view of the boxed area shown in panel A. (B) No discernable immunosignal for TRPC5 could be detected in TRPC1/C4/C5-tko neurons. (C and D) Line-scan analyses (dashed lines shown in panels A and B) confirm the high degree of colocalization between TRPC5 (red) and bassoon (green). (E) TRPC5 colocalizes with bassoon in presynaptic terminals in wt cells. No TRPC5 signal could be detected in tko neurons. The colocalization was determined in thin axonal structures to avoid the contribution of somatic or dendritic TRPC5 staining. Data were collected from wt neurons (19 images, 179 ROIs) and tko neurons (21 images, 168 ROIs) prepared from 3 independent cultures. ***p = 1.5 × 10^−11^, Mann-Whitney rank sum test. Underlying data can be found in S1 Data. ROI, region of interest; tko, triple knockout; TRPC, transient receptor potential canonical; wt, wild type. (TIF) [file pbio.3000445.s001.tif]

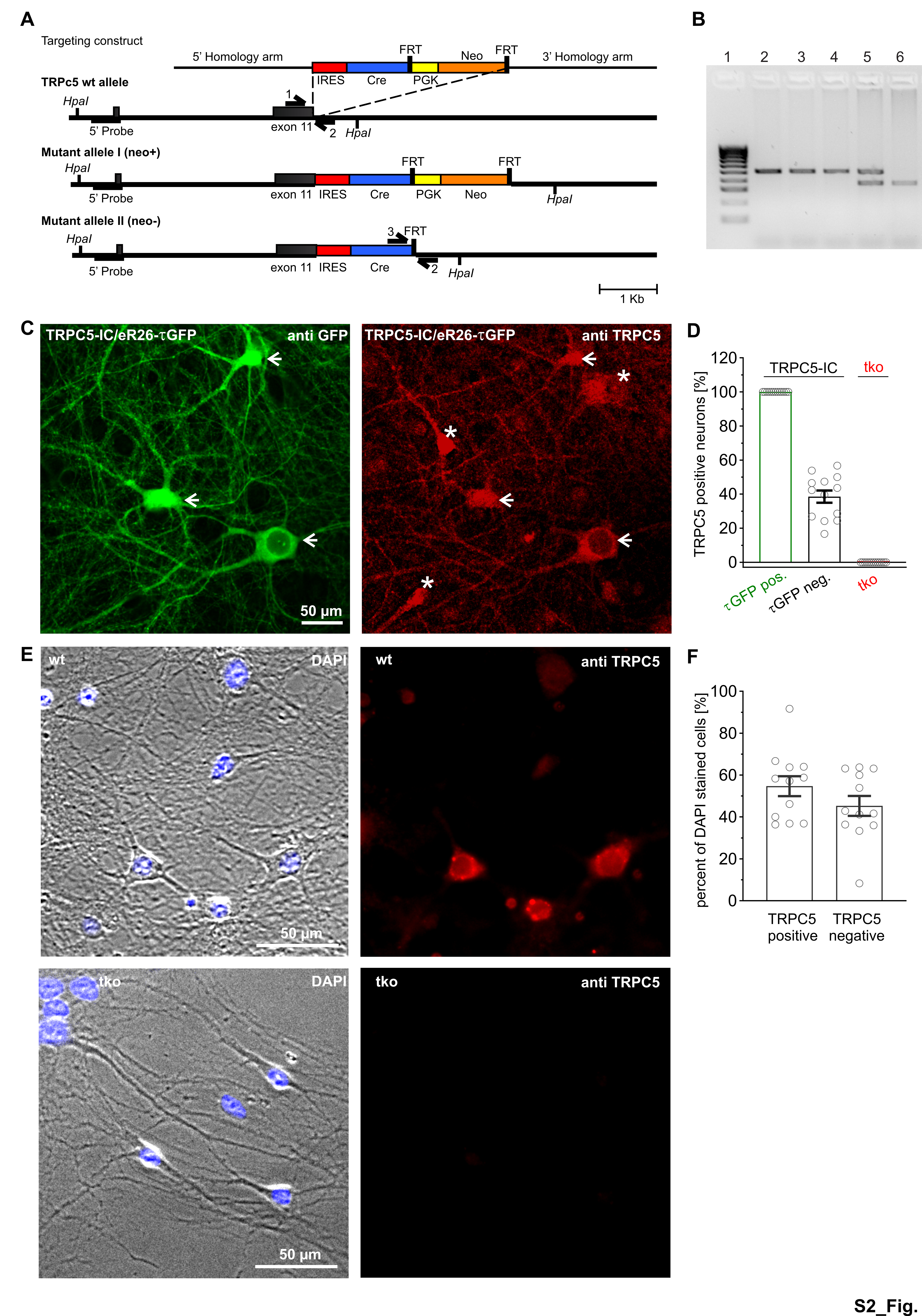

Supplement: S2 Fig — (A) Generation of the TRPC5-IRES-Cre mouse strain. Schematic representation of the targeting strategy used to express Cre recombinase under control of the TRPC5 promoter. From top to bottom, the targeting vector, the TRPC5 wt allele and the targeted TRPC5 allele before (neo+) and after (neo−) removal of the neomycin cassette are shown. The restriction sites for HpaI and the location of the probe are indicated. The inserted cassette is composed of an IRES followed by the coding sequence for Cre recombinase (Cre) and a pgk promoter-driven neomycin selection cassette flanked by FRT sites. (B) Genotyping of TRPC5-IRES-Cre mice. Representative genotyping result from mice already carrying the neoallele, expected product sizes are 364 bp for wt and 500 bp for neoallele. Lanes 4, 5, and 6 represent TRPC5-IRES-Cre neohomozygous, heterozygous, and wt mice, respectively. Sites of primer annealing are shown in panel A; primers 1 and 2 in combination produce the wt band, and primers 3 and 2 produce the TRPC5-IRES-Cre neoband. (C) Confocal images of τGFP immunoreactivity (left panel) and TRPC5 staining (right panel) in hippocampal neurons (12 div) prepared from TRPC5-IC/eR26-τGFP mice. All τGFP-positive neurons (arrows) express TRPC5. Some cells positive for TRPC5 by immunostaining do not show τGFP staining (asterisk), indicating that τGFP-positive neurons represent a subset of TRPC5 expressing cells. (D) τGFP labeling faithfully reports TRPC5 expression, yet 38.5% ± 3.58% of the τGFP-negative cells were found to TRPC5 positive (data were collected from 831 cells, 2 preparations). No discernable immunosignal for TRPC5 was detected in tko neurons (n = 101 cells, 2 preparations). (E) Hippocampal wt neurons stained with DAPI (left panel, overlaid epifluorescence + brightfield channel) and immunolabeled with TRPC5 antibody (right panel). Only a subpopulation of hippocampal neurons (52% ± 3%, 195 out of 375 cells, 3 preparations) is immunopositive for TRPC5. No discernable staining co [file pbio.3000445.s002.tif]

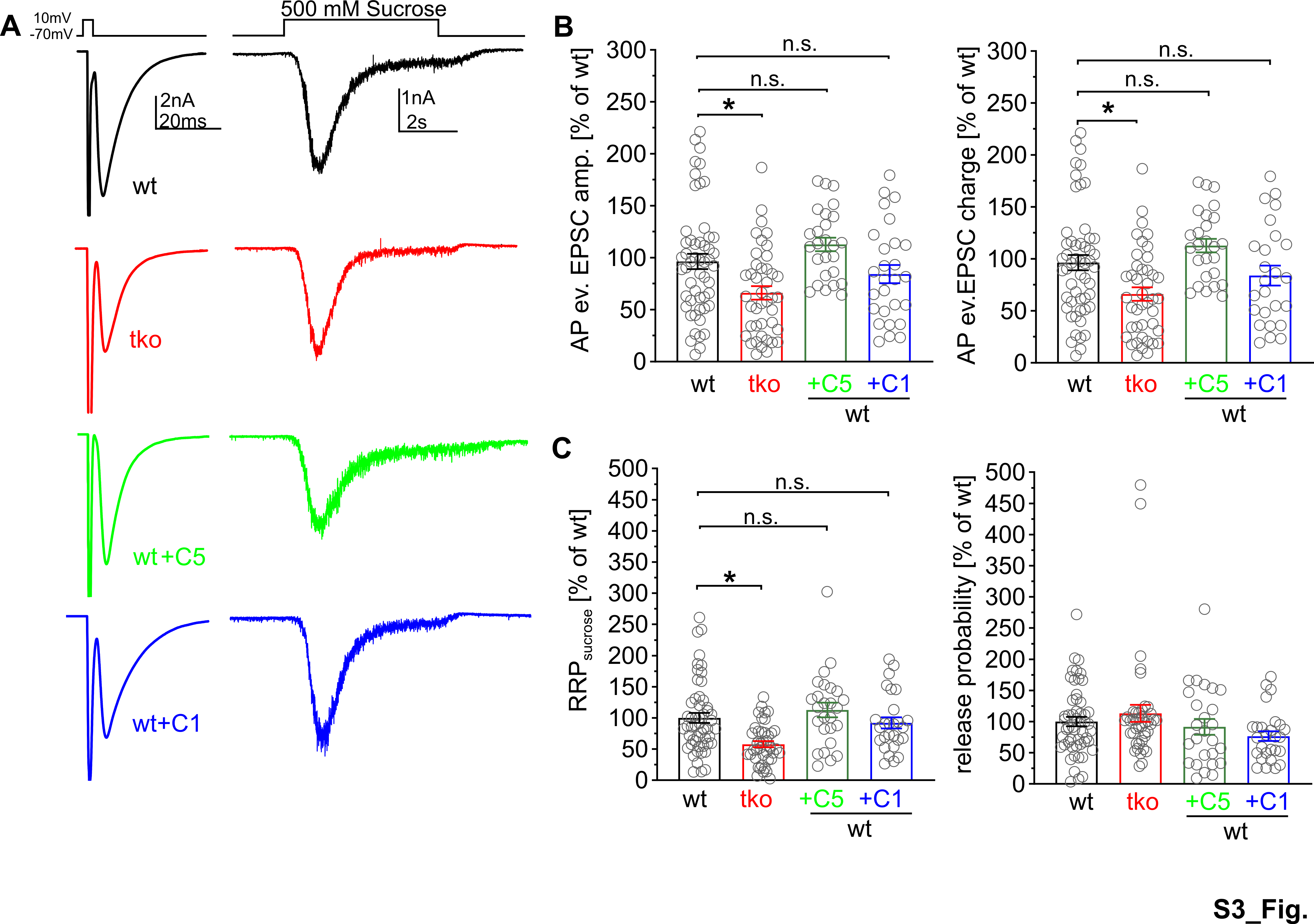

Supplement: S3 Fig — (A) Representative traces of the averaged evoked response (left panel) and the secretory response to 5 s application of 500 mM hypertonic sucrose solution (left panel) for wt, tko, wt + TRPC1, and wt + TRPC5 cells. (B) The evoked EPSC amplitude (left) and EPSC charge (right) are significantly reduced in tko neurons (amp: wt versus tko, p = 0.026; wt versus +C1, p = 0.31; wt versus +C5, p = 1.0; charge: wt versus tko, p = 0.01; wt versus +C1, p = 0.11; wt versus +C5, p = 0.9). (C) The RRP charge (left panel), determined by the time integral over hypertonic response, is reduced in tko cells (wt versus tko, p = 0.0004; wt versus +C1, p = 0.9; wt versus +C5, p = 0.9). The release probability determined by the ratio of EPSCcharge/RRPcharge is unchanged (right panel; wt versus tko, p = 0.9; wt versus +C1, p = 0.2; wt versus +C5, p = 0.9). Data were collected from wt, n = 53; tko, n = 42; wt + TRPC1, n = 27; wt + TRPC5, n = 26; *p < 0.05; one-way ANOVA on ranks followed by Dunn’s post hoc test. Underlying data can be found in S1 Data. EPSC, evoked postsynaptic current; RRP, readily releasable pool; tko, triple knockout; TRPC, transient receptor potential canonical; wt, wild type. (TIF) [file pbio.3000445.s003.tif]

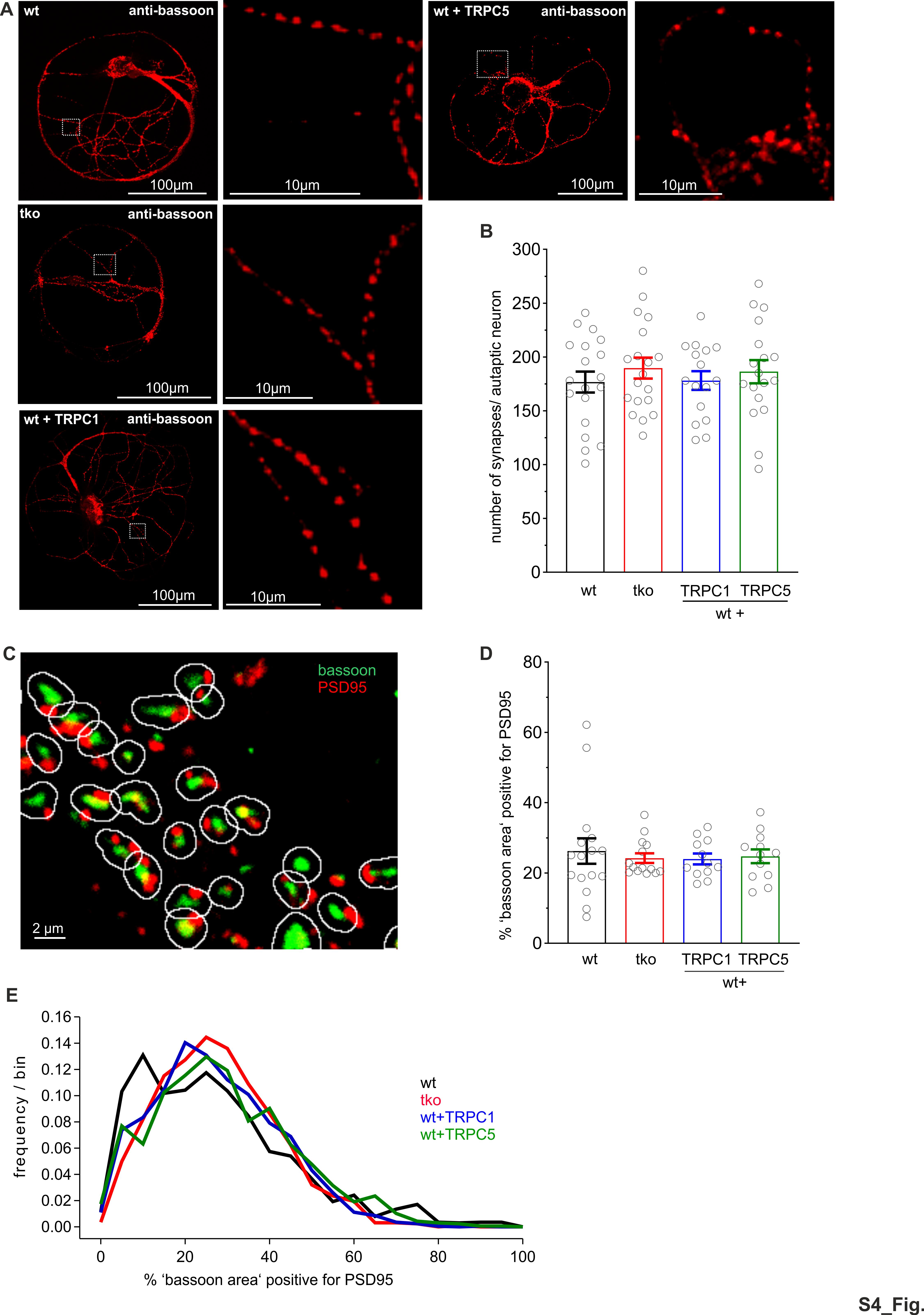

Supplement: S4 Fig — (A) Exemplary confocal images (displayed as maximum intensity projections over 15 sections) of autaptic wt, tko, and wt neurons expressing TRPC1 or TRPC5. Immunolabeling with the presynaptic marker protein bassoon revealed no differences in the number of synapses. (Right panels) Higher magnification of presynaptic terminals from a single confocal section from the corresponding image on the left(dashed box). (B) The number of synapses (identified by counting single bassoon-positive puncta throughout the stack) was unchanged among the different groups (one-way ANOVA on ranks followed by Dunn’s post hoc test, p = 0.742). Data were collected from wt, n = 19; tko, n = 20; wt + TRPC1, n = 18; wt + TRPC5, n = 18. (C) Exemplary confocal image of cultured hippocampal wt neurons immunostained for the active zone protein bassoon and the postsynaptic density protein PSD-95. Bassoon and PSD-95 partially colocalize but more often occupy adjacent domains, consistent with their localization in pre- and postsynaptic compartments. Solid lines define the area wherein the colocalization or apposition of bassoon and PSD95 was quantified. Images are displayed as maximum intensity projection of 3 z-planes. (D) Neither loss of TRPC1/C4/C5 (tko) nor expression of TRPC1 or C5 in wt neurons affects the apposition of bassoon and PSD-95 when compared with controls. Values are given as mean of median determined from the parameter’s frequency distribution for each cell. Data were collected from wt, n = 16; tko, n = 14; wt + TRPC1, n = 12; wt + TRPC5, n = 12; p = 0.985; one-way ANOVA on ranks followed by Dunn’s post hoc test. (E) Mean frequency distributions of the relative “bassoon area” covered by PSD-95 staining for wt, wt neurons expressing TRPC1 or TRPC5, and tko neurons. Statistical power (pr) was determined with a post hoc power analysis. Underlying data can be found in S1 Data. PSD-95, post synaptic density protein 95; tko, triple knockout; TRPC, transient receptor potential canonical; wt, [file pbio.3000445.s004.tif]

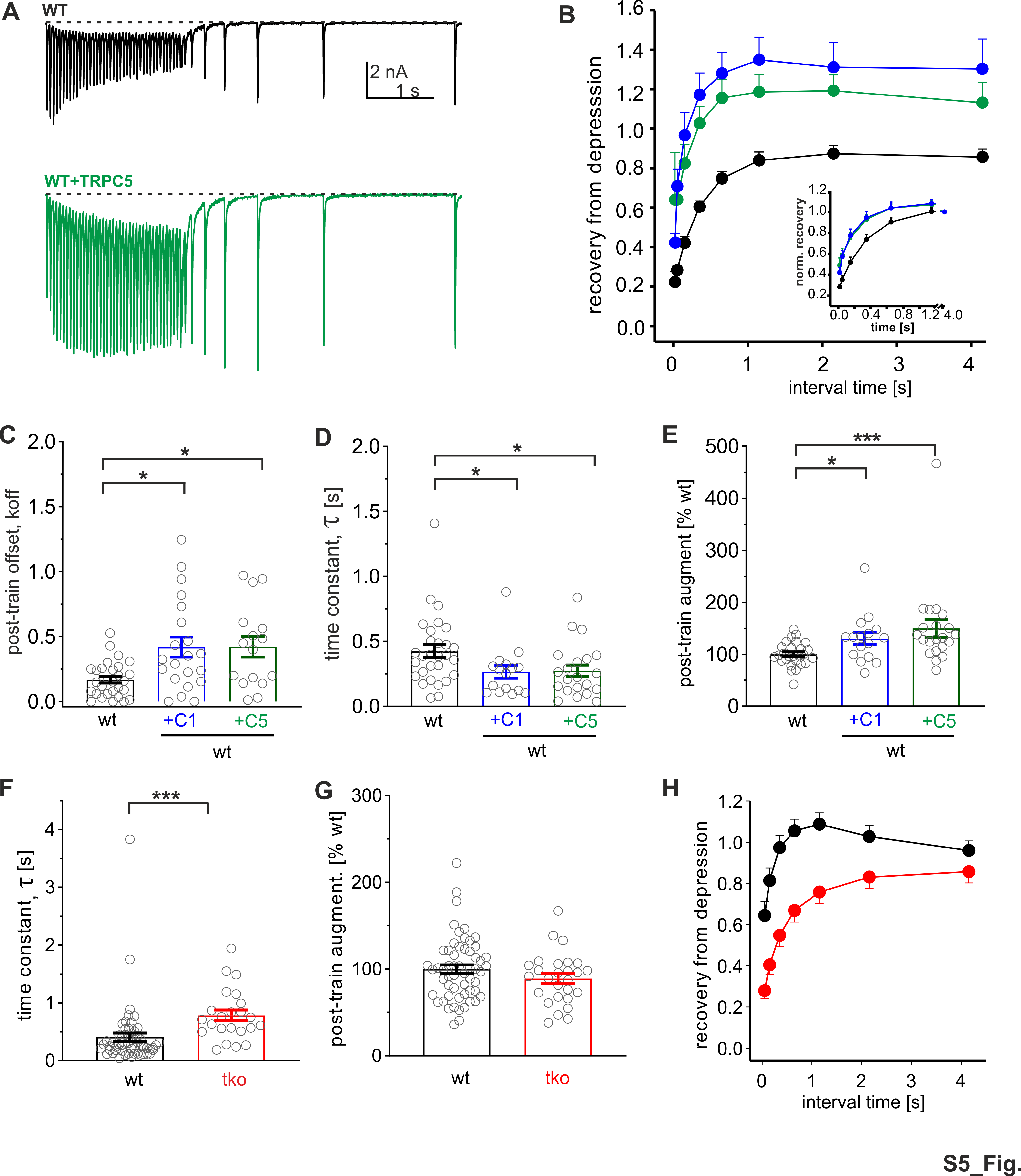

Supplement: S5 Fig — (A) Representative EPSC traces (20 Hz, 2 s) of wt (black) and TRPC5-expressing wt neurons (green). Following HFS, the time course of recovery was determined by test pulses given after 20, 50, 150, 350, 650, 1,150, 2,150, and 4,150 ms intervals. (B) Time course for the recovery of the EPSC amplitude. Fraction of recovery was determined by dividing EPSC amplitudes of the test pulses by the first amplitude of the HF train. Data were fitted with a single exponential function (EPSC(t) = koff + EPSC(∞) × (1-exp-(t/τ)), revealing reduced depression during HFS (koff, C), faster recovery kinetics (τ, D), and transient post-train augmentation of the synaptic response (EPSC(4s), E) with expression of TRPC1 or TRPC5. (Inset) expansion of the early phase of the plot after normalization to the wt response. (C) TRPC1 or C5 expression increases koff because of diminished synaptic depression during HFS (+C5, *p = 0.025; +C1, *p = 0.018; versus wt). (D and E) TRPC1 or TRPC5 expression significantly speeds up the recovery from depression (D; wt versus +C5, *p = 0.039, wt versus +C1, *p = 0.049; one-way ANOVA on ranks followed by Dunn’s post hoc test) and leads to post-train augmentation of the synaptic response (E; +C5, p = 0.0421, wt versus +C1, p = 0.0009, versus wt). Data were collected from wt, n = 29, wt + TRPC1, n = 21, wt + TRPC5, n = 16. (F) tko neurons show a slower rate of recovery from depression. Analysis was restricted to cells with significant depression during HFS to determine the time course of recovery with reasonable fidelity (wt, n = 44; tko, n = 25 cells; ***p = 0.000003; Mann-Whitney rank sum test). (G) The post-train augmentation of the EPSC was not significantly altered for tko neurons. (H) Time course of recovery for wt and tko cells (p = 0.73, Mann-Whitney rank sum test). Loss of TRPC channels slows down the rate of recovery from HFS; data were collected from wt, n = 57; tko, n = 25 cells. Underlying data can be found in S1 Data. EPSC, evoked postsynaptic curr [file pbio.3000445.s005.tif]

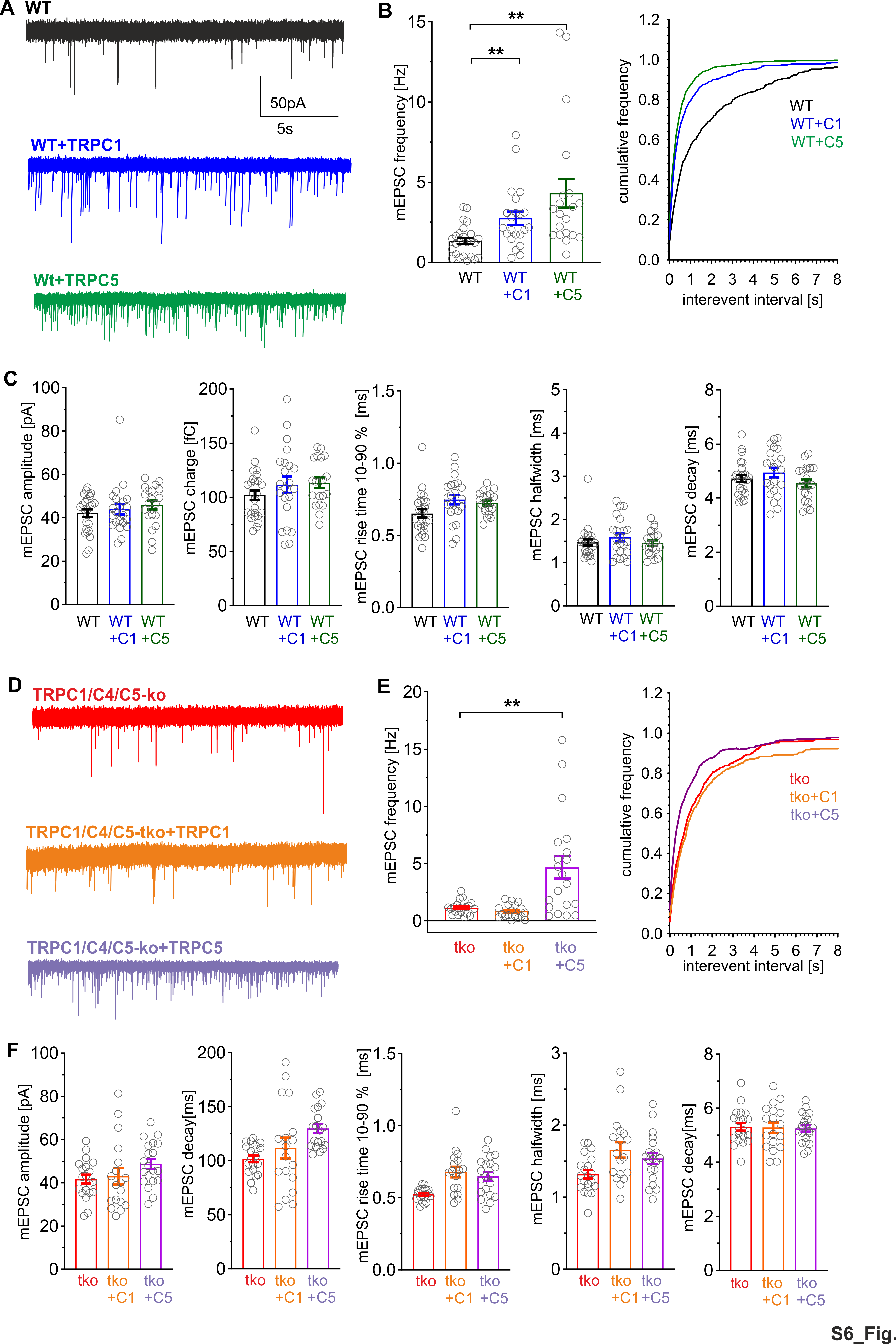

Supplement: S6 Fig — (A) Exemplary recordings of spontaneous mEPSC signaling in neuronal mass cultures recorded in the presence of 10 μ M TTX. (B) Expression of either TRPC1 or TRPC5 significantly increases the mEPSC frequency (left panel). Corresponding cumulative frequency distributions of the mEPSC interevent interval (right panel). Data were collected from wt, n = 25; wt + C1, n = 22; p = 0.006; wt + C5, n = 20; p = 0.0001 cells. (C) The kinetics of the quantal events as illustrated for the indicated parameters remained unaffected by TRPC expression. (D) Sample recordings of mEPSC signaling from tko neurons expressing either TRPC1 or TRPC5. (E) Expression of TRPC5 but not of TRPC1 promotes an increase in mEPSC frequency (left panel). Corresponding cumulative frequency distributions of the mEPSC interevent interval (right panel). (F) mEPSC signaling with respect to amplitude and kinetics remained unchanged. Data were collected from tko, n = 20; tko + C1, n = 18; p = 0.91; tko + C5, n = 20; p = 0.002. **p < 0.01, ***p < 0.001; one-way ANOVA on ranks followed by Dunn’s post hoc test. Underlying data can be found in S1 Data. mEPSC, miniature evoked excitatory postsynaptic current; tko, triple knockout; TRPC, transient receptor potential canonical; TTX, tetrodotoxin; wt, wild type. (TIF) [file pbio.3000445.s006.tif]

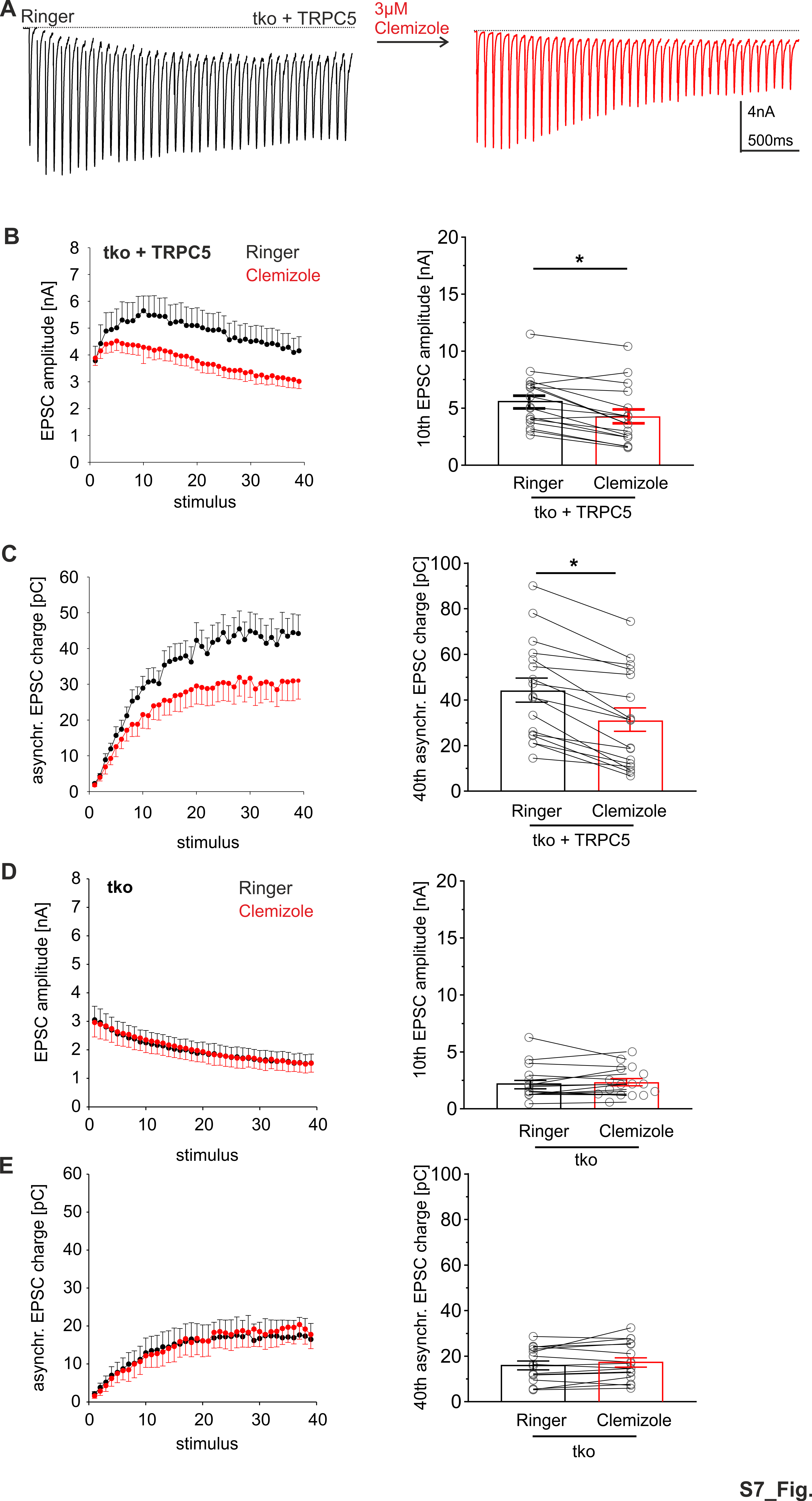

Supplement: S7 Fig — (A) Sample EPSC recordings of TRPC5-expressing tko neurons during 20 Hz HFS before (left) and after application of clemizole (3 μM, 90 s; right). (B) Time course of the EPSC amplitudes during HFS for Ringer and subsequent clemizole application. (Right panel) Clemizole significantly diminishes the EPSC amplitude (10th pulse; p = 0.016). (C) The asynchronous charge (determined for the 40th AP during the train) is significantly decreased with clemizole superfusion (p = 0.031). (D and E) In tko neurons, clemizole application neither affects the EPSC amplitude (10th pulse) nor the asynchronous charge (E). Data were collected from 3 independent preparations, tko, n = 16; tko + TRPC5, n = 17; *p < 0.05; paired t test. Underlying data can be found in S1 Data. AP, action potential; EPSC, evoked postsynaptic current; STE, short-term enhancement; tko, triple knockout; TRPC, transient receptor potential canonical. (TIF) [file pbio.3000445.s007.tif]

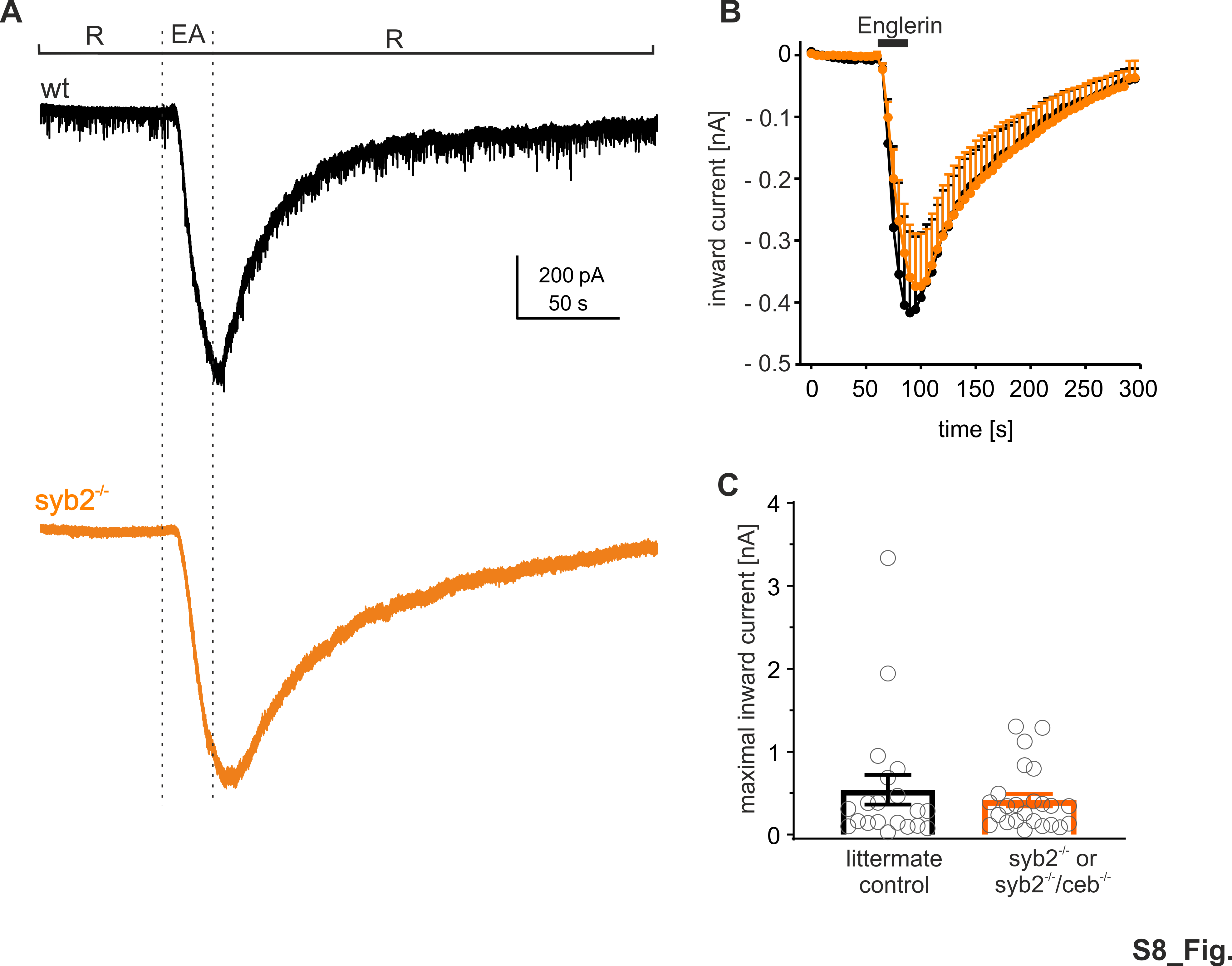

Supplement: S8 Fig — (A) Exemplary recordings of autaptic wt and synaptobrevin2-deficient neurons (syb2-/-) superfused with Ringer (R) solution containing EA (1 μM). No mEPSCs could be recorded in syb2−/− neurons indicative of the exocytotic block upon genetic loss of syb2. (B and C) Neither the time course (B) nor the peak amplitude (C) of the EA-evoked current response was significantly altered in neurons lacking syb2 or in the common absence of syb2 and its homolog cellubrevin (syb2−/−/ceb−/−, dko). Data were pooled from syb2 ko and dko animals because loss of syb2 is crucial for abolishing transmitter release, and no significant differences between the phenotype of the corresponding littermate controls (wt neurons and ceb ko neurons) were detected. Data were collected from littermates (n = 13) and syb−/− or syb−/−/ceb−/− (n = 16); p = 0.983; Mann-Whitney rank sum test. Underlying data can be found in S1 Data. ceb, cellubrevin; dko, synaptobrevin2 and cellubrevin double knock out; EA, Englerin A; ko, knock out; mEPSC, miniature evoked excitatory postsynaptic current; syb2, synaptobrevin2; TRPC, transient receptor potential canonical; wt, wild type. (TIF) [file pbio.3000445.s008.tif]
